# An enormous potential for niche construction through bacterial cross-feeding in a homogeneous environment

**DOI:** 10.1371/journal.pcbi.1006340

**Published:** 2018-07-24

**Authors:** Magdalena San Roman, Andreas Wagner

**Affiliations:** 1 Department of Evolutionary Biology and Environmental Studies, University of Zurich, Zurich, Switzerland; 2 Swiss Institute of Bioinformatics, Lausanne, Switzerland; 3 The Santa Fe Institute, Santa Fe, New Mexico, United States of America; DAL, CANADA

## Abstract

Microorganisms modify their environment by excreting by-products of metabolism, which can create new ecological niches that can help microbial populations diversify. A striking example comes from experimental evolution of genetically identical *Escherichia coli* populations that are grown in a homogeneous environment with the single carbon source glucose. In such experiments, stable communities of genetically diverse cross-feeding *E*. *coli* cells readily emerge. Some cells that consume the primary carbon source glucose excrete a secondary carbon source, such as acetate, that sustains other community members. Few such cross-feeding polymorphisms are known experimentally, because they are difficult to screen for. We studied the potential of bacterial metabolism to create new ecological niches based on cross-feeding. To do so, we used genome scale models of the metabolism of *E*. *coli* and metabolisms of similar complexity, to identify unique pairs of primary and secondary carbon sources in these metabolisms. We then combined dynamic flux balance analysis with analytical calculations to identify which pair of carbon sources can sustain a polymorphic cross-feeding community. We identified almost 10,000 such pairs of carbon sources, each of them corresponding to a unique ecological niche. Bacterial metabolism shows an immense potential for the construction of new ecological niches through cross feeding.

## Author summary

Biodiversity can emerge in a completely homogeneous environment from populations with initially genetically identical individuals. This striking observation comes from experimental evolution of bacteria, which create new ecological niches when they excrete nutrient-rich waste products that can sustain the life of other bacteria. It is difficult to estimate the potential of any one organism for such metabolic niche construction experimentally, because it is challenging to screen for novel metabolic abilities on a large scale. We therefore used experimentally validated models of bacterial metabolism to predict how many novel niches organisms like *Escherichia coli* can construct, if a novel niche must be able to sustain a stable community of microbes that differ in the nutrients they consume. We identify thousands of such niches. They differ in their primary carbon source and a secondary carbon source that is excreted by some microbes and used by others. Because we restricted ourselves to chemically simple environments, we may even have underestimated the enormous potential of microbes for niche construction.

## Introduction

With as many as one trillion predicted species, microbial diversity on our planet is enormous [1]. To understand the origins of biological diversity in general and microbial diversity in particular is a central goal of ecology and evolutionary biology. For many decades, most biological diversity was thought to arise in allopatry, that is, when populations become physically subdivided [2]. More recently, biologists have increasingly accepted that populations can also diversify in sympatry, that is, without any physical barriers [3–9]. Examples of sympatric diversification include insect populations that adapt evolutionarily to different plant hosts [9], stickleback populations that evolve reproductive isolation at least partly in sympatry [6], Midas cichlid populations that originated in a small volcanic crater lake in Nicaragua [7], and bacteriophage lambda that specializes on different bacterial hosts [8]. In bacteria, sympatric divergence has been observed both in nature [10,11] and during experimental evolution [12–15].

Sympatric diversification is easiest in heterogeneous environments [16,17]. Because such environments provide multiple ecological niches, organisms can easily diversify when they specialize and adapt to these niches. Such diversity can then be maintained according to the niche exclusion principle–the principle states that different organisms cannot occupy the same niche [18]. Examples include the spatial structure of an unshaken growth medium, which facilitates morphological diversification in experimental evolution of *Pseudomonas fluorescens* [12]; spatial (free-living or particle-associated) and temporal (spring and fall) resource partitioning, which triggers sympatric speciation in bacterioplankton [19]; the divergence that occurs as a result of host shifts from hawthorn to domestic apples in apple maggot flies [9]; as well as the specialization of bacteriophages to *Escherichia coli* expressing different membrane proteins [8,9].

In apparent contradiction to the niche exclusion principle, sympatric diversification can also occur in homogeneous environments [6,7,10,11,13,14,20]. Perhaps the most striking example involves stable genetic polymorphisms that can originate in *E*. *coli* populations cultured in the homogeneous and well-mixed environment of a batch culture or a chemostat, a device in which a cell culture is kept in a constant nutrient environment by continually supplying it with nutrient medium [13,14,20–23]. For example, over a mere 800 generations of laboratory evolution in a glucose-limited chemostat, initially isogenic populations of *E*. *coli* can diversify into multiple genetically different strains [13,21–23]. These strains stably coexist in the chemostat as a result of cross-feeding [22].That is, one strain consumes the primary carbon source glucose and excretes a secondary carbon source (acetate or glycerol), whereas the other strain feeds on the secondary carbon source. These phenotypic differences result from regulatory DNA mutations in transcription factors and cis-regulatory regions. They include a cis-regulatory mutation affecting the expression of acetyl CoA synthetase, an enzyme that catalyzes the transformation of acetate to acetyl CoA, which enters the tricarboxylic acid cycle to produce energy. They also include a structural mutation in the glycerol-3-phosphate repressor, which can result in constitutive expression of glycerol utilization genes [21]. Experiments like this suggest that *E*. *coli* may readily diversify genetically and metabolically in a completely homogeneous environment.

The emergence of cross-feeding is an example of niche construction, a process where organisms change their environment in ways that can affect the evolutionary dynamics of themselves and of other organisms [24–29]. Prominent examples of niche construction include animals that construct artifacts such as webs, nests and burrows [24]; earthworms and plants that alter the fertility, humidity and chemical composition of soil [28,30,31]; and bacteria that construct biofilms and excrete antibiotics as well as metabolic by-products [32]. Constructed niches can affect evolution even on the short time scales of experimental evolution, where populations of *Pseudomonas fluorescens* become dependent on their own modifications of their chemical environment [33,34].

The origin of new niches associated with bacterial cross-feeding is not easy to detect experimentally: Except for differences in colony morphology, cross-feeding polymorphisms generally lack phenotypes that are both macroscopically visible and highly specific. However, computational analysis can help predict the conditions under which cross-feeding polymorphisms can originate and persist. Some authors use small biochemical networks to search for the conditions that promote genetic diversification through cross-feeding interactions [35–40]. Others use digital organisms with evolvable genomes and metabolic networks [41]. Yet others simulate individuals in an evolving population where random mutations can change nutrient consumption rates in a model of *E*. *coli* central carbon metabolism, and show that glucose-acetate cross-feeding can originate in such a population. Most recently, a genome scale metabolic network of *E*. *coli* was used to study cross-feeding and other metabolic dependencies that emerge as a result of evolution under gene loss [42] or amino-acid leakage [43].

Here we go beyond this work and evaluate the general potential for the construction of new niches associated with cross-feeding that is inherent to the metabolism of *E*. *coli* and to complex metabolic systems in general. That is, we ask how many different kinds of ecologically stable cross-feeding interactions can emerge in an initially homogeneous population, where one bacterial strain feeds on a primary carbon source and produces a secondary carbon source that sustains the other strain. To answer this question, we take advantage of a well-studied and experimentally validated [44] genome-scale model of *E*. *coli* metabolism. We use Flux Balance Analysis (FBA), an experimentally validated computational technique [45], to characterize the production of secondary carbon sources that can help cross-feeding polymorphisms emerge. We then use dynamic flux balance analysis (dFBA) [46,47], a variant of FBA that uses genome-scale metabolic information to predict the ecological dynamics of microbial communities and how they change their chemical environment over time. We use dFBA to study the conditions under which two cross-feeding strains can establish a stable community in a chemostat. After having reproduced the experimentally observed glucose-acetate cross-feeding polymorphism [13], we then identify additional pairs of primary and secondary carbon sources that can lead to the establishment of stable cross-feeding communities. We find thousands of such pairs, both in *E*. *coli* and other metabolic reaction networks of similar complexity. Our work demonstrates the great potential of metabolic systems to construct new ecological niches.

## Results

### The model

Our first analysis prepares the ground by examining the conditions under which a glucose-acetate cross-feeding polymorphism can be stably maintained by two *E*. *coli* strains. We studied this specific polymorphism, because it is experimentally well documented [13,21–23], and aimed to reproduce it *in silico*. Specifically, we simulated the dynamic of a community composed of two cross-feeding *E*. *coli* strains (or ecotypes [48]), a *producer* strain P that produces a secondary carbon source as a by-product of feeding on some primary carbon source, and a consumer strain C that consumes this secondary carbon source. We use the same genome scale metabolic network of *E*. *coli i*JO1366 [44] to model both strains. This means that the metabolic networks of both strains comprise exactly the same reactions and metabolites. This modeling decision reflects the observation that cross-feeding strains can emerge from a single *E*. *coli* ancestor in little evolutionary time [22]. The metabolic differences between cross-feeding strains do not result from differences in their complement of enzyme-coding genes, but from regulatory mutations that affect how much of a specific carbon source each strain can consume or produce [49].

We model these differences phenomenologically, through differences in the flux through two specific reactions in strains P and C. Specifically, we model the secondary carbon source production of strain P by imposing a non-zero production flux *p*_*scs*,*P*_ for this carbon source via the exchange reaction that transports the secondary carbon source out of the cell. And we model the secondary carbon source consumption of strain C by limiting the strain’s primary carbon source consumption. This modeling decision is motivated by the experimental observation that when cross-feeding emerges in *E*. *coli* [22], the consumer strain’s ability to consume its primary carbon source becomes impaired. One might argue that increasing the consumption of the secondary carbon source might be biologically more sensible. However, the two approaches are equivalent. Here is why. Since we simulate a chemostat culture, once steady state is reached, the strains in the chemostat grow at a constant rate. The consumer strain C achieves this growth rate by consuming both primary and secondary carbon sources. If consumption of the primary carbon source increases, consumption of the secondary carbon source becomes reduced by an equivalent amount (Eq 3, S4 Text), such that the steady state is unaffected.

For the well-studied cross-feeding interaction of acetate producer and consumer strains, regulatory mutations in specific genes are known to bring forth the metabolic behavior of producer and consumer strains [21,23,50]. Since the objective of our work was to study not only glucose-acetate cross-feeding but multiple other cross-feeding interactions we decided not to incorporate assumptions about specific mutations in specific genes into our model. By imposing general constraints on the production and consumption of specific carbon sources, we allowed for the possibility that our modeled strains could achieve these constraints in different ways, depending on the carbon source considered. The specific mutations that may underlie our strains’ metabolic behavior will be the subject of future work.

### Acetate production creates a two-dimensional ecological niche that can stably support two *E*. *coli* strains through cross-feeding

**Fig 1:**
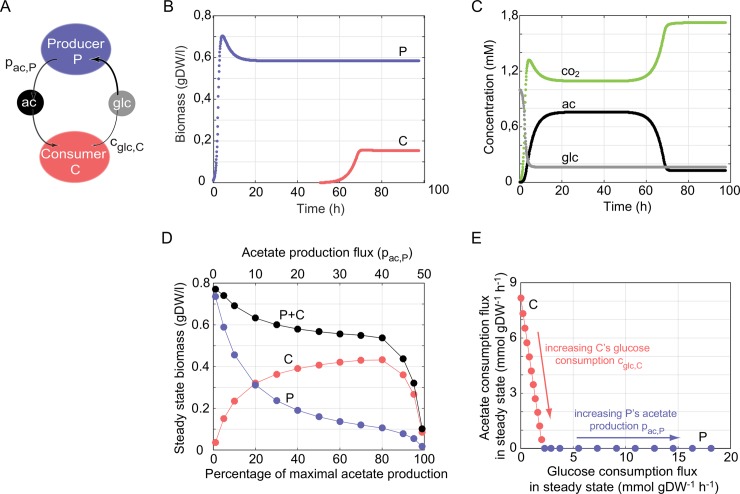
Ecological dynamics of an acetate producer *E*. *coli* strain P and an acetate consumer strain C in a chemostat. (A) Interactions between the strains. Producer strain P (blue) produces acetate (black) at a rate *p*_*ac*,*P*_ and consumes glucose (grey) as its sole carbon source. Consumer strain C consumes mainly acetate but can also consume glucose at some rate *c*_*glc*,*C*_. Both strains may also consume other nutrients or produce other metabolic by-products, which are not shown. (B) Dynamics in a chemostat for an acetate production flux by P of 2.6 (*p*_*ac*,*P*_ = 2.6 mmol∙gDW^-1^h^-1^). The horizontal axis shows time, and the vertical axis shows the biomass of P (in blue) and C (in red) vs. time. (C), as in (B), but the vertical axis shows the concentration of glucose (grey), acetate (black) and carbon dioxide (green). (D) Steady state biomass (vertical axis) of the producer strain (P, in blue), consumer strain (C, in red), and both strains (P+C, in black) as a function of the acetate production rate (horizontal axis). This rate is expressed either in absolute flux units (top horizontal axis) or as the percentage of the maximal acetate production rate (bottom horizontal axis), that is, the rate beyond which the producer strain grows so slowly that it is flushed out of the chemostat. (B), (C) and (D) show the results of simulations of ecological dynamics in a chemostat inhabited by an acetate producer strain P and an acetate consumer strain C. For the purpose of these figures, it is assumed that strain C cannot consume glucose (*c*_*glc*,*C*_ = 0). (E) Nutritional niche of the producer strain P (blue) and the consumer strain C (red) when metabolically distinguishable strains coexist. The horizontal and vertical axes show the glucose and acetate consumption rates, respectively, of the indicated strains in metabolic steady-state. P’s steady-state nutrient consumption increases with its acetate production *p*_*ac*,*P*_ (blue arrow). The blend and ratio of nutrients that C consumes varies with the maximal glucose consumption rate *c*_*glc*,*C*_. That is, increasing C’s maximal glucose consumption *c*_*glc*,*C*_, increases C’s glucose consumption in steady state while reducing its acetate consumption, as indicated by the red arrow.

In the first part of our analysis, we focus on glucose as a primary carbon source, and on acetate as a secondary carbon source. The secondary carbon source is excreted by the producer strain P at a rate *p*_*ac*,*P*_, and consumed by the consumer strain C (Fig 1A). We initially assume that the consumer strain C cannot consume glucose (*c*_*glc*,*C*_ = 0), an assumption that we relax below (S3 Text). To find out whether both strains can coexist in a stable chemostat community, we first use Flux Balance Analysis [45](FBA, Methods) in the form of dynamic FBA [46,47] (Methods).

To mimic typical experimental conditions, we performed all simulations with a dilution rate *D*, the rate at which culture is replaced with fresh medium, of *D* = 0.2 h^-1^[13]. At this dilution rate, the maximum rate at which *E*. *coli* cells can produce acetate ($p_{ac}^{max}$) without being eventually flushed out from the chemostat is 50.3 mmol gDW^-1^ h^-1^ (S2 Text). (Here and below, all units of metabolic flux are given in mmol gDW^-1^ h^-1^). To ensure survival of the producer strain P, we simulated chemostat dynamics at an acetate production rate *p*_*ac*,*P*_ that is equal to 5% of this maximum (2.6 mmol gDW^-1^ h^-1^). We initialized the chemostat in the presence of only the acetate producing strain P, and once this strain had reached steady-state, which occurred after no more than 50 hours, we introduced the acetate consuming strain C. We then monitored the joint dynamics of both strains until they had reached steady-state or until one strain had gone extinct.

Fig 1B shows the change in biomass of P and C over time. Only three carbon-containing metabolites–glucose, acetate, and carbon dioxide–change their concentration (Fig 1C).The concentration of glucose (Fig 1C, grey) decreases as the acetate producer P consumes glucose and this decrease is concurrent with an increase in P's biomass (Fig 1B, blue). Strain P metabolizes glucose partially to carbon dioxide (Fig 1C, green) and partially to acetate (Fig 1C, black), which is why the concentration of both metabolic by-products increases. Once the acetate consumer strain C is introduced into the chemostat at 50 hours (Fig 1B, red), the chemical environment contains a substantial amount of acetate, which strain C metabolizes to carbon dioxide to synthesize biomass. By 100 hours, the system has reached a new steady state, in which a stable polymorphism of the acetate producer (P) and the acetate consumer (C) strain is maintained as a result of their cross-feeding interaction.

We next wanted to find out how the population’s behavior changes if the amount of acetate excreted by the producer strain P varies. We thus varied the acetate production rate *p*_*ac*,*P*_ up to the maximum beyond which the producer goes extinct. Not surprisingly, the steady-state biomass of strain P is reduced as its acetate production increases (Fig 1D, blue), because of the metabolic cost incurred by acetate production. In contrast, the steady-state biomass of the consumer strain C has a unimodal distribution, with a maximum biomass reached at approximately 80% of the maximal acetate production rate. The reason is that C's biomass reflects the acetate concentration in the chemostat, and this concentration depends not only on the amount of acetate produced per unit of producer strain (*p*_*ac*,*P*_), but also on the amount of producer biomass. As acetate production *p*_*ac*,*P*_ increases, the amount of acetate produced per unit biomass increases but the amount of producer biomass decreases. The joint effect of these opposing patterns is a unimodal distribution of consumer biomass.

The total (community's) biomass (Fig 1D, black) decreases with increasing acetate production and has its maximum (0.78 gDW/l) in the absence of acetate production. The reason is that part of the acetate excreted into the chemostat environment is removed through the dilution flux D and not available for usage. In addition, even if all produced acetate were available, its production and later consumption are associated with losses in terms of energy and carbon atoms.

Regardless of the acetate production of the producer strain P, the producer and consumer strains stably coexist, as has also been found experimentally [22]. Additional simulations show that the eventual steady-state composition of the chemostat does not depend on the initial biomass of either strain or the time at which C is introduced (S1 Fig). In contrast, a higher dilution flux D will result in higher biomass for the producer P, but a lower biomass for the consumer C. This can be intuitively understood if we consider the concentration of the nutrients that support growth of each strain: The higher the dilution rate is, the more similar the composition of the chemostat is to that of the fresh medium, which contains high amounts of glucose but no acetate.

When the consumer strain C can also metabolize the primary carbon source (*c*_*glc*,*C*_ > 0), the two strains compete for this carbon source, and coexistence is no longer guaranteed. However, analytical calculations supplemented by simulations show that the two strains can stably coexist under a broad range of glucose consumption and acetate production rates (S3 and S4 Texts, S2 and S3 Figs). When they do, they occupy distinct ecological niches in their nutrient environment [51,52], as shown in Fig 1E. One can visualize the ecological niche space in our chemostat environment as a multidimensional space, where each axis of the space corresponds to the availability or consumption of a nutrient available in the environment. Because in our analysis only two carbon sources are present, the producer strain P and the consumer strain C can compete only for these two carbon sources, which renders our niche space two-dimensional (Fig 1E). Its axes correspond to glucose and acetate consumption rates in metabolic steady state. The ecological niches for our two strains can overlap at the level of glucose consumption (Fig 1E and S3 Fig), but the strains cannot consume identical amounts of glucose without losing their metabolic differences. Thus, their ecological niches cannot overlap completely, consistent with the competitive exclusion principle from ecological theory [18]. We note that this conception of a niche is consistent with the geometric framework of nutritional niche representations [51,52], where niches correspond to the “blend and ratio of nutrients that maximize fitness”.

### Growth on glucose can create multiple additional carbon-source niches

**Fig 2:**
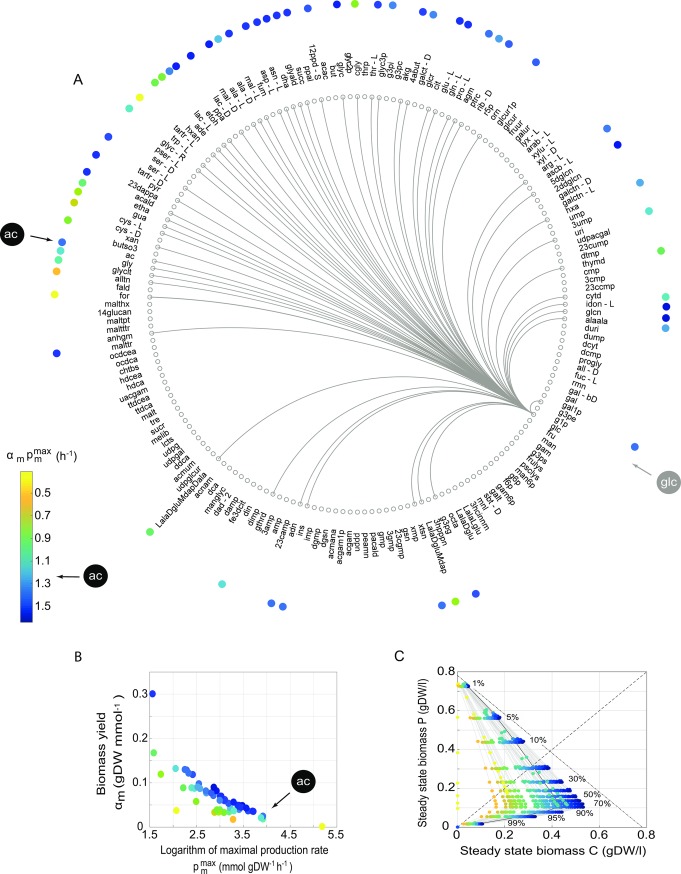
Multiple possible cross-feeding interactions involving glucose as a primary carbon source. (A) Each of the 180 small grey circles labeled with an acronym corresponds to a carbon source that can sustain viability of *E*. *coli* (*i*JO1366) when present as a sole carbon source. Metabolites are ranked by increasing biomass yield, starting with formate at 9’ o’clock. Arcs connect glucose (glc, grey arrow) with a carbon source (circles) m, if m can be produced when *E*. *coli* grows on glucose as the sole carbon source. The black arrow indicates the location of the secondary carbon source acetate (ac). Colored circles near each secondary carbon source represent the product of maximal production ($p_{m}^{max}$) and biomass yield (*α*_*m*_) of the carbon source. The magnitude of this product is represented by color (color bar), and this color encoding is applied to all three panels of the figure. (B) Biomass yield and maximal production flux for each secondary carbon source on glucose. The black arrow indicates the circle corresponding to acetate. (C) Steady state biomass values of producer strain P (vertical axis) and consumer strain C (horizontal axis) at different percentages of the maximum synthesis rate ($p_{m}^{max}$), i.e., the synthesis rate of the secondary carbon source beyond which the producer strain P is flushed out of the chemostat, for all secondary carbon sources (grey lines). Circles are placed at 1, 5, 10–90, 95 and 99% of the maximum synthesis rate $p_{m}^{max}$, as indicated by the numbers in the panel. The dashed-dotted line indicates the total steady-state biomass that is maximally achievable (0.78 gDW/l, obtained when glucose is metabolized completely to CO_2_ without synthesis of any secondary carbon source). The dashed line indicates where both strains have identical biomass. The product of maximal production ($p_{m}^{max}$) and biomass yield (*α*_*m*_) equals 1.26 h^-1^ for acetate (black line superimposed with blue circles).

Our analysis so far reproduced the experimentally observed construction of the glucose-acetate niche [14,22], and identified the conditions under which two *E*. *coli* strains can coexist in this niche (Fig 1D, S3 and S4 Texts, S2 and S3 Figs). We next turn to secondary carbon sources other than acetate. Although only glycerol has been experimentally identified as an additional secondary carbon source in cross-feeding experiments [14,22], *E*. *coli* cells can produce many other metabolites when growing on glucose [53]. These metabolites, as well as possibly additional, still unknown metabolites, might serve as secondary carbon sources. To identify all possible secondary carbon sources, we first identified all carbon containing metabolites in the *i*JO1366 metabolic network that can be transported across the cell wall (i.e., metabolites containing an associated exchange reaction). We used FBA to identify which of these molecules can sustain *E*. *coli* growth when present as the sole carbon source, which is a prerequisite for a molecule’s usefulness in the cross-feeding interactions we study. FBA predicts 180 metabolites (whose acronyms are given in the circle of Fig 2A) that can sustain growth of *E*. *coli* when used as sole carbon sources (Methods). (See Supplementary Table 3 in [44] for a list of standard metabolite acronyms used in Fig 2A).

For each of these 180 metabolites, we determined whether *E*. *coli* can produce the metabolite when it is provided with glucose as the sole carbon source (See methods). Fig 2A shows a graphical representation of the answer, where an arc connects glucose (grey arrow) to another carbon source if that carbon source can be produced when glucose is the sole primary carbon source. (This means that the enzymes needed to transform glucose into the carbon source are present in *E*. *coli*). There are 58 such secondary carbon sources, acetate (black arrow) being one of them. In other words, *E*. *coli* cells growing on glucose can modify the environment by producing 58 alternative nutrients, each of which can sustain the life of other *E*. *coli* individuals.

The secondary carbon sources differ greatly in the maximum rate ($p_{m}^{max}$) at which they can be produced (Fig 2B), which ranges from 4.75 mmol gDW^-1^ h^-1^ for N-Acetyl-D-glucosamine(anhydrous)N-Acetylmuramic acid to 178 mmol gDW^-1^ h^-1^ for formate. Acetate’s maximal synthesis rate is 50.3 mmol gDW^-1^ h^-1^, about twice the mean production rate of 24.7 mmol gDW^-1^ h^-1^, and second highest among all secondary carbon sources (together with glycolate). This maximum production rate reflects the cost of producing a carbon source: The costlier the production of a secondary carbon source is, the smaller is its maximum production rate (S4 Fig). In addition, the secondary carbon sources differ in their specific biomass yield *α*, which is the growth rate that can be achieved per unit of carbon source consumed (see Fig 2B and S2 Text and S7 Fig green dots). This yield varies from 0.0014 to 0.30 (in gDWmmol^-1^of carbon source). Acetate’s biomass yield equals 0.025 and is thus low, less than half of the mean value of 0.068. Thus, while acetate is not costly to produce, its low biomass yield also does not allow for much biomass production in strains that consume it. (See S1 File for biomass yields, maximum production rates, and production costs for all secondary carbon sources).

Following our analysis of the glucose-acetate niche (Fig 1), we then asked whether glucose, in combination with each of these individual metabolites, could lead to a stable cross feeding polymorphism. In other words, are there values of the production rate of metabolite *x* (*p*_*x*,*P*_) and the consumption rate of glucose (*c*_*glc*,*C*_) that lead to a stable cross-feeding polymorphism? We found that all secondary carbon sources other than formate can sustain a stable community of two strains, even though these communities vary greatly in the amount of total biomass that they contain (Fig 2C).

If strain P completely respires glucose to carbon dioxide and thus does not excrete any secondary carbon source, the total community biomass is equal to the biomass of P, and reaches a maximum value of 0.78 gDW/l, which is indicated by a dashed-dotted line in Fig 2C. For any one secondary carbon source excreted by strain P, the steady-state biomass of the community will change with the carbon source’s excretion rate, up to a sustainable maximum ($p_{m}^{max}$) beyond which the producer grows so slowly that it will eventually be flushed out from the chemostat. The figure indicates this change by one grey line (and superimposed colored circles) for each of the 54 secondary carbon sources, along with percentages that indicate the percentage of the maximum rate $p_{m}^{max}$ at which strain P produces the secondary carbon source. (Displaying secondary carbon source excretion as a percentage of this allowable maximum has the advantage of displaying the same cost for all producers, regardless of which secondary carbon source they produce, such that at any given percentage of this maximum, producers of all secondary carbon sources reach the same steady-state biomass.) As the amount of a secondary carbon source produced by P increases, the total community biomass decreases, i.e., the circles in Fig 2C become further removed from the dashed-dotted line. We already observed this behavior for acetate (Fig 1C, black line in Fig 2C), but Fig 2C illustrates that it holds for all secondary carbon sources.

The absence of a stable community in the glucose-formate niche space is a consequence of formate’s low biomass yield, which requires high formate consumption (112 mmol gDW^-1^ h^-1^) to support a growth rate greater than the dilution rate D = 0.2 h^-1^. This level of consumption is impossible under our assumed transport limit (*V*_*max*_ = 20 mmol gDW^-1^ h^-1^). Higher transport limits or lower dilution rates would, however, permit the existence of a stable community.

The steady-state biomass changes of both strains P and C with increased production of a secondary carbon source (Fig 2C) are analogous to what we observed in Fig 1D, and they exist for the same reason. To support higher production fluxes of any secondary carbon source, P needs to consume more glucose and therefore reaches lower steady-state biomass. To understand the change in steady-state biomass of strain C with an increasing production rate of the secondary carbon source, one has to take into account two factors. The first is the maximal production rate of the secondary carbon source (*p*^*max*^), which affects the carbon source’s availability for C’s consumption. The second is the biomass yield *α* of the secondary carbon source, which affects the growth rate achieved per unit flux of consumed carbon source. The product of production and yield ($\alpha_{m}p_{m}^{max}$) determines the steady-state biomass of C. If a metabolite is costly (with low maximal production) one can expect low excretion, but a high biomass yield of the same metabolite may compensate for its low production and permit a higher steady-state biomass of C. The colors in Fig 2 indicate the magnitude of $\alpha_{m}p_{m}^{max}$ for all 58 secondary carbon sources. The figure illustrates that secondary carbon sources whose product of production and yield ($\alpha_{m}p_{m}^{max}$) is low will lead to communities with lower total biomass.

The product of production and yield $\alpha_{ac}p_{ac}^{max}$ for acetate does not have an unusually large value (equals 1.26 h^-1^). About half of the secondary carbon sources (29 out of the 58) have a higher maximal growth rate $\alpha_{m}p_{m}^{max}$ than acetate ($\alpha_{m}p_{m}^{max}$ > 1.26, blue dots in Fig 2D), and therefore support higher community biomass.

The observations in Fig 2D are based on the assumption that strain C consumes only the secondary carbon source, but not the primary carbon source glucose (*c*_*glc*,*C*_ = 0). However, relaxing this assumption to *c*_*glc*,*C*_ > 0 also allows for stable coexistence of P and C (S6 Fig). The key difference is that stable coexistence then becomes possible for each of the 58 secondary carbon sources, including formate. If C’s glucose consumption is so high that it covers at least 90% of the energy and carbon required for persistence in the chemostat, formate can supply the remaining energy and carbon needed. In this case, coexistence of a formate producer strain and a glucose-formate consumer strain would be possible.

### Primary carbon sources different from glucose can help construct even more novel niches

Because glucose is not the only primary carbon source that can sustain *E*. *coli*, we extended the previous analysis of searching for secondary carbon sources from glucose to all 180 primary carbon sources. We began by identifying the number of potential secondary carbon sources that can be produced when *E*. *coli* grows on each primary carbon source. This number ranges from 54 to 62, depending on the primary carbon source (blue circles in S7 Fig and S8A Fig).

Most secondary carbon sources can be produced from all primary carbon sources, as is the case for acetate, but some secondary sources can be produced from just a few primary carbon sources (red circles in S7 Fig and S8B Fig).

Our analytical results (S4 Text) reveal that coexistence is possible for each pair of primary and secondary carbon sources, as long as two conditions are met. The producer strain must produce the secondary carbon source, and the consumer strain C must be able to persist in the chemostat by consuming both the primary and the secondary carbon source (not just the primary carbon source alone). If this were not the case, that is, if the consumer strain was able to persist in the chemostat by consuming only the primary carbon source, then it would have an advantage over the producer strain, which uses part of the consumed primary carbon source to produce the secondary carbon source. In this case, the producer strain would go extinct.

In total, our analysis finds 83 different secondary carbon sources and 9913 unique pairs of primary and secondary carbon sources that allow stable coexistence of a producer strain P and a consumer strain C (Table 1). Taken together, these observations imply that the synthesis of by-product metabolites by *E*. *coli* can create an enormous number of new ecological niches whose identity depends on the primary carbon source available in the environment.

**Table 1:** Metabolic characteristics of *E*. *coli*, *B*. *subtilis*, *S*. *cerevisiae* and the pan-metabolic network.

	*E*. *coli*	*B*. *subtilis*	S. *cerevisiae*	Pan-metabolic network
Reactions	2583	1250	1577	7222
Metabolites	1805	990	1226	5625
Exchange reactions	330	229	164	330
Primary carbon sources	180	119	52	221
Secondary carbon sources (on glucose)	58	35	31	86
Secondary carbon sources (total)	83	46	34	109
Pairs of primary-secondary carbon sources	9913	4146	1585	18959
Blocked reactions	227	291	553	3070

### The potential for metabolic niche construction is not a peculiarity of *E*. *coli* metabolism

The metabolism of any one organism is the product of a long evolutionary history. *E*. *coli’*s enormous potential for the creation of novel metabolic niches could be an accident of this evolutionary history, or it could be a more general property of the chemical reaction networks that constitute a metabolism. To find out, we performed several additional analyses. First, we analyzed the niche construction potential of two microbes different from and not closely related to *E*. *coli*, i.e., the soil bacterium *Bacillus subtilis* (model *i*YO844 [54]) and the yeast *Saccharomyces cerevisiae* (model *i*MM904, [55]). (See methods for a detailed description of the procedure.) The analysis revealed (Table 1) that these organisms also have a large niche construction potential. They can form stable cross-feeding communities with more than 1000 pairs of primary and secondary carbon sources (Table 1).

Each of these three organisms has its own evolutionary history which molded its metabolic network. The observation that they all share a large potential to construct new metabolic niches hints that this potential is a general property of metabolic systems, and not just a peculiarity of the organisms studied and their evolutionary history. To exclude the influence of this history more rigorously, we repeated our analysis with metabolic networks that are not the product of evolution, but that we created *in silico* with an algorithm that produces *random viable* networks. These are biochemical reaction networks that produce all essential biomass molecules in a given chemical environment, but contain an otherwise random complement of biochemical reactions drawn from the known “universe” of such reactions. We obtained these networks through a previously published [56,57] Markov Chain Monte Carlo (MCMC) procedure that samples such networks from a vast space of metabolic networks (See Methods).

We note that simpler sampling methods, such as “brute force” uniform sampling of a given number of reactions from a reaction universe is very unlikely to yield viable networks [56]. In contrast, MCMC sampling can yield not only viable networks but viable networks whose reaction complement is effectively random beyond the requirements imposed by viability, as shown by previous work [56,58].

We used this method to create samples of 500 random viable networks viable on glucose as a sole carbon source and that have the same number of reactions (2251) as the *E*. *coli* network. We made these networks permeable to all 330 metabolites to which *E*. *coli* is permeable. In other words, these random viable metabolisms have the potential to consume and produce the same metabolites as *E*. *coli*.

**Fig 3:**
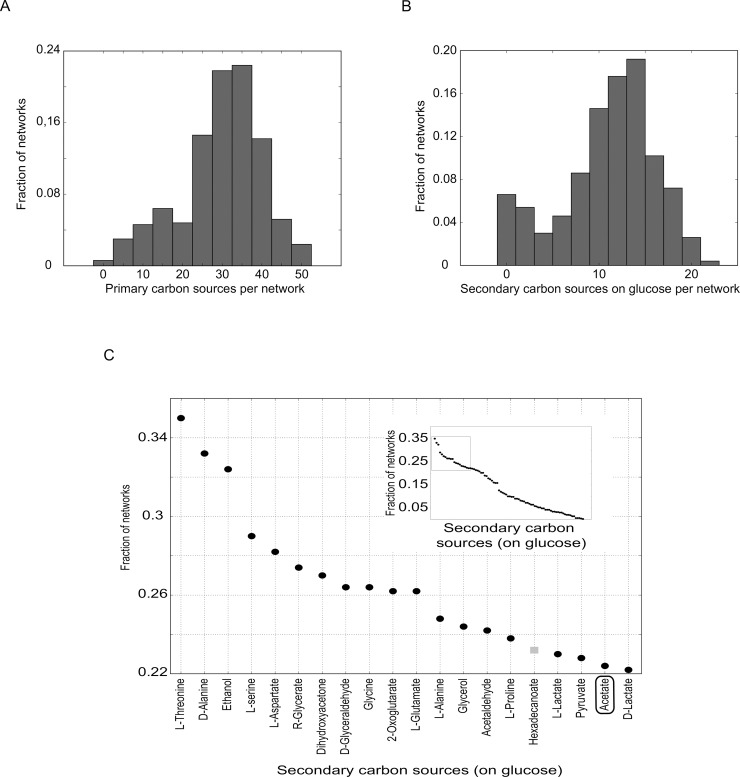
Niche construction potential with generic metabolic systems. (A) Histogram of the number of primary carbon sources on which random metabolic networks required to be viable on glucose are also viable. (B) Histogram of the number of secondary carbon sources produced by random metabolic networks required to be viable on glucose. (C) Rank plot of secondary carbon sources produced by at least one random viable network when glucose is used as a primary carbon source, ranked by the fraction of random viable networks by which the secondary carbon source is synthesized. The main panel shows the names of the 20 metabolites with the highest rank, which includes acetate (black box) and glycerol. The grey square shows one secondary carbon source that occurred in random viable networks but not in *E*. *coli*. The inset shows all 84 secondary carbon sources that are produced by at least one random network viable on glucose.

In our sampling procedure, we only required these networks to be viable on glucose, but as a result of complex correlations between metabolic phenotypes [59,60], they are usually also viable on additional primary carbon sources (Fig 3A). Specifically, the number of primary carbon sources on which each sampled metabolic network is viable ranges from 1 to 52 (mean 32±10) (Fig 3A). We observe 218 primary carbon sources on which at least one of these random networks is viable.

When exposed to glucose as the primary carbon source, these networks produce between 0 and 22 secondary carbon sources (mean 11±5) that can sustain a two-strain community (Fig 3B). Taking all sampled metabolic networks we find 84 secondary carbon sources that are produced by at least one random viable network (26 more than produced by *E*. *coli*). Most secondary carbon sources are produced by more than one random viable network. Fig 3C shows all secondary carbon sources that are produced by any random network, ranked by the fraction of the 500 random viable networks that produce them. Acetate and glycerol, the secondary carbon sources found experimentally when growing *E*. *coli* on glucose are among the top-ranked carbon sources, with respective ranks of 19 and 13.

When exposed to not just glucose but to each of its primary carbon sources in turn, a random viable metabolic network can produce on average 14±6 secondary carbon sources (ranging from 0 to 26). The number of primary-secondary carbon source pairs varied greatly between networks, ranging from 0 to 1065 (mean 404±236). In total we observe 15685 different primary-secondary carbon source pairs that could serve as the foundation of a stable community in at least one random viable network.

In sum, because even random viable metabolisms show high niche construction and cross-feeding potential, this potential is likely an intrinsic property of metabolic systems.

### Niche construction potential in the pan-metabolic network

Our last analysis complements the previous analysis by constructing a pan-metabolic network that contains all metabolic reactions from a known and curated “universe” of metabolic reactions (Methods). For various reasons, such a network could never be realized in any one organism, but it provides another way to inform us which kind of cross feeding interactions are metabolically possible. (S10 Fig illustrates how this pan-metabolism analysis relates to our previous analysis of random viable networks.) The pan-metabolic network we analyzed comprises 7222 reactions and 5625 metabolites. As in our analysis of random viable metabolic networks, we only allowed those 330 metabolites to enter and leave a cell that can also enter or leave *E*. *coli*. This focuses our analysis on novel biosynthetic abilities rather than on novel transport as a reason for the production or consumption of novel carbon sources. It also implies that we may underestimate the numbers of primary and secondary carbon sources, and perhaps dramatically so.

The pan-metabolic network harbors 221 metabolites that can be used as primary carbon sources, 41 more than *E*. *coli*. The minimum number of secondary carbon sources produced per primary carbon source is 85 (S8C Fig), compared to the 54 in *E*. *coli* (S8A Fig). In total, the pan-metabolic model can produce 109 secondary carbon sources. The total number of primary-secondary carbon source pairs almost doubles relative to *E*. *coli* (18959 and 9913 pairs for the pan-metabolic and the *E*. *coli* network, respectively). See Table 1.

In sum, the numbers of primary-secondary carbon source pairs that can sustain stable communities is greatest in the pan-metabolic network. In different organisms, different subsets of such pairs may be suitable for cross-feeding induced niche construction.

## Discussion

When an isogenic bacterial population grows in a homogeneous environment with a single nutrient or carbon source, the organisms in the population initially behave similarly and consume this nutrient. They may also excrete by-product metabolites that accumulate in the environment. If they can express the necessary enzymes, they may switch to consume the by-products once most of the initial nutrient is consumed. In such a population, DNA mutations may arise that alter metabolic properties like enzyme activities or expression permanently. As a result, ancestor and mutant strains may compete for the original nutrient, and one of them may eventually be excluded from the population. Alternatively, a mutant may specialize in the consumption of the ancestor’s by-products. Our work focuses on this commensal or mutualistic scenario, which can help ancestor and mutant to coexist stably, and thus permanently increase genetic and metabolic diversity.

We searched exhaustively for by-product or secondary carbon sources that can be excreted when a microbial strain grows on some primary carbon source, and that can themselves sustain microbial life. We performed this search with the metabolism of three non closely related organisms: *E*. *coli*, *B*. *subtilis* and *S*. *cerevisiae*; with 500 randomly sampled metabolic networks that we required to be viable on at least glucose and that contained the same number of biochemical reactions as *E*. *coli*; and with a pan-metabolic network containing 7222 biochemical reactions known to occur in extant organisms. For each of these metabolisms we identified thousands of possible cross-feeding interactions where one strain produces a carbon source that can sustain the other strain. Through a combination of analytical calculations and simulations of the ecological dynamics of two-strain chemostat communities, we demonstrated the existence of 9919 unique cross-feeding niches in *E*. *coli* alone that can sustain a stable two-strain community. Each niche corresponds to a unique pair of primary and secondary carbon sources. Our observations suggest an enormous potential for population diversification through niche construction and cross feeding.

Although it may seem puzzling that an organism would dispose of metabolites that could advance its own growth, it is not an unusual phenomenon. The causes are multiple, and include membrane leakage, overflow metabolism, genetic mutations, and cells fermenting carbon sources even in the presence of oxygen [36,61,62]. In addition to acetate, for example, *E*. *coli* frequently releases formate, lactate, succinate and ethanol into the environment as a result of fermentation or membrane leakage [61]. Various microorganisms, including *Escherichia coli*, *Corynebacterium glutamicum*, *Bacillus licheniformis* and *Saccharomyces cerevisiae*, excrete a broad diversity of more than 30 metabolic intermediates and amino acids [53]. Detecting such secondary carbon sources may promote the experimental discovery of new cross-feeding interactions.

Our work differs in various ways from previous studies on microbial metabolic interactions that include competition, commensalism and mutualism [47,63–71] in general, and cross-feeding in particular [35–42,72–74]. The most closely related studies [42,47,63] use a metabolic model of *E*. *coli* to study the various cross feeding interactions that can emerge in co-culture after single gene knock-out [63] or extensive gene loss [42]. Our work, in contrast, shows that even without such alterations to its reaction complement *E*. *coli* can create many niches. In addition, we also analyzed other organisms, as well as random viable metabolisms to demonstrate that this niche construction potential is not just a property of *E*. *coli* or closely related organisms, but a generic property of complex metabolic systems. Other authors have demonstrated that microbes from different species that are cultured together can show new biosynthetic abilities [47]. In contrast, our work shows that new niches and stable communities can emerge from within a population of initially identical individuals. And perhaps most importantly, we have not merely reproduced a single experimentally demonstrated niche construction process, but found that metabolic systems can give rise to myriad new niches through cross-feeding.

Our analysis has several limitations. First, we rely on current knowledge about the metabolism of *E*. *coli*, *B*. *subtilis*, *S*. *cerevisiae* and on reactions in the pan-metabolic network. Future research is likely to discover additional reactions in these networks. They may allow the consumption of additional primary carbon sources, or the synthesis of additional secondary carbon sources. In either case, such additional reactions can only increase, not decrease, the niche construction potential of metabolism.

Second, whereas different organisms can import or excrete a different spectrum of molecules, our analysis of random viable networks and the pan-metabolic network allowed only those metabolites to enter or leave a cell that can also enter or leave *E*. *coli*. Even so, we found thousands of potential niches. Had we opened cellular transport to further molecules, the number of niches would have increased as well and perhaps dramatically so.

Third, we varied only carbon sources. Similar analyses could be conducted for sources of other chemical elements, such as nitrogen or sulfur. Again, the potential for niche construction could only increase in this case, because different sources of a chemical element can facilitate to the production of novel secondary metabolites. In sum, these limitations, when overcome, would strengthen our conclusion.

Fourth, random viable metabolic networks and the pan-metabolic network may contain thermodynamically infeasible ATP producing cycles [75–77] that can alter biomass growth. For this reason, it would not have been sensible to simulate cross-feeding dynamics for these metabolic networks. However, our analytical calculations show that the conditions for coexistence hold generally and independently of any one metabolism.

Our observations raise the question why the only known cross-feeding polymorphisms that have been detected in *E*. *coli* chemostats involve acetate and glycerol as secondary carbon sources. One candidate reason is that many other such polymorphisms exist but have not been detected, because currently no systematic screen for cross-feeding interactions exists. Cross-feeding polymorphisms are usually manifest in different colony morphologies on agar plates, and substantial biochemical and genetic work is needed to prove that such polymorphisms result from cross feeding [13,14,22]. A second candidate reason is that in many such polymorphisms, one of the strains may constitute a small fraction of community biomass, which would make its detection even harder. For instance, we showed (Fig 2D) that half of the secondary carbon sources that *E*. *coli* can produce in a glucose environment cause a high metabolic cost to the producer strain or little biomass gain to the consumer strains, which leads to an even lower biomass of the consumer strain than for glucose-acetate cross-feeding. Third, perhaps not all cross-feeding polymorphisms we predict can be biologically realized. For example, on some primary carbon sources multiple regulatory mutations may be needed before a strain produces or consumes some of the secondary carbon sources we predict. Even though such combinations of mutations may arise in large populations of bacteria, the respective secondary carbon sources will be less frequently produced than carbon sources for which single mutations suffice. Characterizing the regulatory mutations needed to bring forth specific secondary carbon sources is a complex undertaking that we will focus on in future work.

Our work focuses on bacterial populations, but similar phenomena may occur elsewhere. For example, they may help explain a hallmark of cancer, the metabolic heterogeneity within tumors [78]. Many tumors occupy low oxygen-environments, because they grow faster than blood vessels can form. As a result, they synthesize fermentation products like fumarate or succinate [79]. In addition, even when oxygen is available, tumor cells exhibit the Warburg effect [80], the fermentation of glucose to lactate. It is possible that these phenomena may help create new nutritional niches that may be colonized by tumor cells.

Like most biodiversity, bacterial diversity may have arisen through repeated adaptive radiations, in which a single lineage rapidly diversifies to occupy multiple ecological niches [12,81–83]. Usually, species created during adaptive radiations are thought to occupy pre-existing niches, but the rapid emergence of extensive cross-feeding in homogeneous environments [13,14,20] raises the possibility that many niches are constructed during a radiation. That is, when a bacterial population excretes one or more energy-rich by-product metabolites, it creates niches that can be occupied by mutant strains that are well-adapted to these niches. By excreting their own specific metabolites, these strains can then become stepping stones towards further diversification. In this process, the new metabolic niches into which a population radiates are constructed by the population itself. Because any one bacterial strain can excrete a broad spectrum of metabolites, and because our work identified thousands of niches that could sustain stable communities, the potential for such diversification should not be underestimated. We hope that our observations will motivate experimental work that identifies the extent to which this potential is realized.

## Methods

### Flux balance analysis (FBA)

Flux balance analysis (FBA) is a computational method to predict metabolic fluxes–the rate at which chemical reactions convert substrates into products–of all reactions in a genome-scale metabolic network [45]. FBA requires information about the stoichiometry of chemical reactions in a metabolic network. It makes two central assumptions. The first is that cells are in a metabolic steady-state. The second is that cells effectively optimize some metabolic property such as biomass production (growth). Additional constraints can be incorporated into the optimization problem that FBA solves, in order to account for the thermodynamic and enzymatic properties of a network’s biochemical reaction. The optimization problem that FBA solves can be formalized as a linear programming problem [45,46] in the following way:

$$\mathrm{Maximize}\;v_{growth}$$

s.t.Sv=0

$$l_{i}\leq v_{i}\leq u_{i}$$

Here, *S* is the stoichiometric matrix, a matrix of size *m* × *r* that mathematically describes the stoichiometry of the network’s metabolic reactions. The integer *m* denotes the number of metabolites and *r* denotes the number of biochemical reactions in the network. These reactions include all known metabolic reactions that take place in an organism, which are called internal reactions. They also include reactions that represent the exchange (import or export) of metabolites with the external environment. Furthermore, they include a biomass growth reaction, which is a “virtual” reaction that reflects in which proportion biomass precursors are incorporated into the biomass of the modeled organism [44–46]. Each entry *S*_*ij*_ of the stoichiometric matrix contains the stoichiometric coefficient with which metabolite *i* participates in reaction *j*. The vector *v* is a vector (of size *r*) that harbors the metabolic flux through each reaction in the network. *v*_*growth*_ specifies the flux through the biomass growth reaction. Fluxes through biochemical reactions are restricted by lower and upper bounds that constrain the flux through each reaction in the network. These bounds are given by the variables *l* and *u*, respectively, which are vectors of size *r*. We performed FBA optimization with the GNU Linear Programming Kit (GLPK; http://www.gnu.org/software/glpk).

### The dynamics of producer strain P and consumer strain C in a chemostat

Strains P and C will grow at rates *μ*_*P*_ and *μ*_*C*_, a process that will increase their respective biomasses *X*_*P*_ and *X*_*C*_. In a chemostat, fresh medium is continuously added and culture is continuously removed at a *dilution rate D*. Such dilution leads to a decrease in biomass inside the chemostat. Overall, the change in biomass for P and C can be expressed by the following system of ordinary differential equations:

$$\frac{dX_{P}}{dt}=(\mu_{P}-D)X_{P}$$ (1)

$$\frac{dX_{C}}{dt}=(\mu_{C}-D)X_{C}$$ (2)

The concentration of any one metabolite (*M*) in a chemostat also varies. If a metabolite is present in the fresh medium at concentration *M*^0^, the metabolite's concentration will increase at a rate *DM*^0^ as a result of fresh medium continually being added to the chemostat. In addition, the metabolite’s concentration will also increase if it is produced by strain P (with flux $J_{M,P}^{out}$) or C (with flux $J_{M,C}^{out}$). The total rate at which *M* is produced will then equal $J_{M,P}^{out}X_{P}\Delta t+J_{M,C}^{out}X_{C}\Delta t$. Conversely, the concentration of *M* will decrease due to removal of old medium at a rate *DM*, and possibly also due to consumption by P (with flux $J_{M,P}^{in}$) and C (with flux $J_{M,C}^{in}$), at a total rate $J_{M,P}^{in}X_{P}\Delta t+J_{M,C}^{in}X_{C}\Delta t$. We denote the net flux of metabolite *M* as $J_{M}=J_{M}^{out}-J_{M}^{in}$, i.e., which results in a positive net flux *J*_*M*_ if the metabolite is produced and negative otherwise. Overall, the change in concentration for each metabolite present in the chemostat is then described by the differential equation

$$\frac{dM}{dt}=D(M^{0}-M)+J_{M,P}X_{P}+J_{M,C}X_{C}$$ (3)

We performed FBA to compute instantaneous growth rates (*μ*_*P*_ and *μ*_*C*_) in h^-1^), as well as consumption and excretion fluxes of each metabolite by strains P and C (*J*_*M*,*P*_ and *J*_*M*,*C*_, in mmol gDW^-1^ h^-1^). We used the values thus computed in dynamic FBA [46] to determine the changing amounts of biomass (expressed in *gDW*/*l*) of our microbial strains, as well as the abundance of all metabolites (in *mM*), nutrients, and waste products in our simulated chemostat.

### Simulating chemostat dynamics with dynamic FBA (dFBA)

Dynamic FBA (dFBA) [46] is an FBA-based method to describe the temporal growth dynamics of microbes and how this dynamics affects the microbes’ chemical environment. It has been used, for example, to describe chemical growth and by-product secretion of *E*. *coli* in batch and fed-batch cultures [46], to study the dynamics of a two-species microbial ecosystem in batch culture [47] and to simulate the growth and metabolic dynamics of microbes in time and space [84].

Briefly, dFBA starts from some initial time point and performs Flux Balance Analysis (FBA) iteratively at each time point during a given time interval to compute the maximally possible growth rate for each strain in the chemostat environment. As microbial strains grow, they consume nutrients and excrete waste products (including, possibly, secondary carbon sources) and thus change their growth environments. Dynamic FBA takes these changes into account by computing the chemical composition of the environment at each time point. In doing so, dynamic FBA predicts how the biomass of bacterial strains and the chemical composition of the environment can change over time.

We used dFBA to predict the temporal behavior of a microbial population composed of acetate producer strain P and consumer strains C in a chemostat. We next describe in detail how we used dFBA to simulate population growth in a glucose-limited minimal medium. We note that our procedure can be applied to any other carbon source by substituting glucose with the desired carbon source.

Our simulations used the following parameters and initial conditions. We chose a dilution rate of D = 0.2 h^-1^ to mirror conditions from previous experiments that had identified cross-feeding interactions [13]. We set the glucose concentration in the fresh medium to 1 mM, close to the 0.7 mM used in [13]. We assumed that ammonium, calcium, chloride, cobalt, copper, iron, magnesium, manganese, molybdate, nickel, oxygen, phosphate, potassium, protons, sodium, sulphate and zinc are present in non-limiting amounts. The initial concentrations of all metabolites in the chemostat are identical to those of the fresh medium. Unless otherwise stated, we initialized the chemostat with 0.01 gDW/l of strain P and 0.001 gDW/l of strain C which corresponds to an overall cell density of approximately 10^7^ cells/ml [85–87]. We chose these initial biomass values arbitrarily, except that their unequal values are well-suited to ask whether the consumer strain C, when introduced in small amounts into a culture of strain P, can invade the culture. However, we also show that changing initial biomass values, the ratio of the biomass values, and the time of introduction of C into the chemostat have no effect on the biomass of P and C once steady-state is reached (S1 Fig).

After initializing our simulations, using these parameters, we discretized time into short intervals of 0.1 h and performed dynamic FBA [46] by iterating the following three steps (described in more detail below): calculation of maximum nutrient uptake rates, FBA, and calculation of environmental composition.

*1*. *Calculation of maximum uptake rates*. The uptake of a nutrient by an organism is limited by two factors: the capacity to transport the nutrient across the cell wall (*transport limitation*) and the availability of the nutrient in the environment (*nutrient availability limitation*). To determine the nutrient transport limit (for a nutrient at concentration *M*), we assumed Michaelis-Menten kinetics (*V*_*max*_*M*/(*k*_*M*_ + *M*)) with parameter values set to *V*_*max*_ = 20 mmol gDW^-1^ h^-1^ and *k*_*M*_ = 0.05 mM. These parameters are based on data in the Brenda enzyme database [88,89] and have been used in related analyses [47].

At the beginning of a simulation, the nutrient concentration is high and the biomasses of P and C are low. Therefore, nutrient consumption is initially limited by transport. As biomass grows the nutrient begins to be scarce and nutrient availability rather than transport become limiting for nutrient consumption. In other words, the transport limit shapes the transient biomass dynamics but the availability limit determines the steady-state biomass. This also means that *V*_*max*_ and *k*_*M*_ can vary over a wide range without affecting the steady state. In S1 Fig we demonstrate the chemostat dynamics for various values of *V*_*max*_ and *k*_*M*_ to exemplify how these parameters modify the transient biomass dynamics, but not the steady state.

To determine the nutrient availability limit, we divided the nutrient concentration *M* by the nutrient consuming biomass. This biomass depends on how much nutrients the strains consumed in the immediately past (according to Eqs (1) and (2) in S1 Text). We describe and justify our procedure to calculate the nutrient availability limit, which differs from that of some other authors, in depth in the supplementary material (S1 Text).

Once we had determined a strain’s transport limit for a nutrient and the nutrient’s availability limit, we set the uptake rate of the nutrient to the minimum of both, which ensures that organisms do not consume more of a nutrient than is physiologically feasible and available to them.

*2*. *FBA*. Once we had calculated maximum uptake rates of nutrients as just described, we performed FBA for each strain independently. The calculation yielded growth rate values (*μ*_*P*_ and *μ*_*C*_) for both strains, as well as consumption or excretion rates of each metabolite M for both strains (*J*_*M*,*P*_ and *J*_*M*,*C*_).

*3*. *Calculation of environmental composition*. With the results of FBA in hand, we used Euler’s method [90] to determine the environmental change caused by nutrient consumption, waste production, and biomass growth. We did so in accordance to Eqs (1), (2) and (3), using the conditions from the beginning of this section and a time increment of 0.1 h.

We repeated these three steps until at most 1000 h (10^4^ time steps) had elapsed or until the chemostat had reached steady state. We assumed that steady state had been reached if the standard deviation of growth rates determined over 50 consecutive time steps was smaller than 10^−5^ for both strains. We carried out these simulations using MATLAB (Mathworks Inc.).

### Search for primary and secondary carbon sources

We searched for all metabolites that could serve as carbon sources in the following way. To identify primary carbon sources, we first considered all metabolites in the *E*. *coli* model *i*JO1366 [45] a candidate primary carbon source, if it contained at least one carbon atom and if *E*. *coli* had an exchange reaction for this carbon source. Second, we used FBA to determine *E*. *coli*’s maximal biomass production when each of these primary carbon sources was available as the sole carbon source. (We assumed that ammonium, calcium, chloride, cobalt, copper, iron, magnesium, manganese, molybdate, nickel, oxygen, phosphate, potassium, protons, sodium, sulphate and zinc can be consumed without constraints). Third, if any one carbon source was able to sustain non-zero biomass production, we considered it an actual primary carbon source. Here and below, we viewed only biomass production fluxes above 10^−5^ mmol gDW^-1^ h^-1^ as being different from zero.

Our approach identified 180 primary carbon sources (Fig 2A). On about half of these carbon sources, growth of *E*. *coli* has been demonstrated experimentally [91,92]. No experimental data is available for multiple other carbon sources. Metabolic reconstruction errors may account for the discrepancies between computational predictions and experimental observations for some other carbon sources, but at least for the well-studied *E*. *coli*, they may be a minor cause compared to regulatory constraints that are not incorporated by most genome-scale models analyzed with FBA [92]. Such regulatory constraints, where enzymes are encoded by a genome but are not expressed when needed, can be easily broken. That is, even on the short time scales of laboratory evolution, microbial populations can adapt to grow on a novel carbon source in accordance with FBA predictions [93]. Because regulatory evolution can occur during the long-term cultivation of *E*. *coli* that we model, we assume that regulatory constraints can be by-passed, and thus use all 180 primary carbon sources on which FBA predicts growth in our analyses.

We considered a metabolite a secondary carbon source if (i) it can serve as a primary carbon source and (ii) if it can be produced as a metabolic by-product when another metabolite serves as a primary carbon source. The first condition ensures that the metabolite can sustain growth of a strain consuming it, and the second condition ensures that the metabolite can be produced. Note that all primary carbon sources are *potential* secondary carbon sources, but only some of them may be produced as metabolic by-products in a given environment. Most importantly, whether a carbon source is produced depends on the available primary carbon source. To identify actual secondary carbon sources and distinguish them from potential ones, we iterated through all pairs of primary carbon sources and potential secondary carbon sources, and performed FBA. More specifically, we used the primary carbon source as the sole carbon source (uptake rate: 10 mmol gDW^-1^h^-1^), maximized the production of the potential secondary carbon source, and constrained biomass production in FBA to be greater than zero. If the potential secondary carbon source could be produced at a rate greater than zero under this constraint, we considered the carbon source an actual secondary carbon source.

We used the same method described in the previous paragraphs to search for primary and secondary carbon sources in the genome scale metabolic networks of *B*. *subtilis* (model *i*YO844 [54]) and *S*. *cerevisiae* (model *i*MM904, [55]), modifying only the chemical environment. Specifically, for *B*. *subtilis* we used an environment composed of ammonium, calcium, carbon dioxide, iron, magnesium, oxygen, phosphate, potassium, protons, sodium and sulphate. We constrained the ammonium, phosphate and sulphate uptake rates of *B*. *subtilis* to a maximum of 5 mmol gDW^-1^h^-1^. For *S*. *cerevisiae*, we used a medium consisting of ammonium, iron, oxygen, phosphate, potassium, protons, sodium and sulphate, and constrained the *o*xygen uptake rate to a maximum of 2 mmol gDW^-1^h^-1^. We obtained all three metabolic models (*i*JO1366, *i*YO844 and *i*MM904) from the BiGG Database[94].

### Pan-metabolic network

The pan-metabolic network is a network containing all metabolic reactions with well-defined stoichiometry that are known to take place in some organism. For our analysis, we extended a previously used pan-metabolic network comprising 5484 metabolites and 6892 reactions [59] by adding the 141 metabolites and 330 reactions from *E*. *coli i*JO1366 that were not already present in this network. This amended pan-metabolic network includes 5625 metabolites and 7222 reactions.

We found that 3070 reactions (43%) in the pan-metabolic network are unconditionally blocked [95]. That is, they cannot carry non-zero flux without violating FBA’s steady state assumption when all metabolites to which *E*. *coli* is permeable can freely enter and leave the cell. We note that if more metabolites where allowed to enter and leave the pan-metabolic network the number of blocked reactions would decrease. For reference, in the well-curated *i*JO1366 model 227 reactions (9%) are unconditionally blocked. In terms of absolute numbers, the pan-metabolic network contains 1569 more reactions that can carry nonzero flux than the *i*JO1366 model.

Genome scale metabolic network reconstructions often contain spurious energy producing cycles that violate the second low of thermodynamic [75–77]. Unless removed from the network, these cycles can spuriously increase biomass production. (For example, removal of these cycles causes an approximately 25% reduction of biomass production in 92% of the networks analyzed in [77]). The pan-metabolic network that we use contains reactions that create spurious ATP producing cycles, allowing ATP to be produced even in the absence of nutrients. However, because 67 metabolites in addition to ATP must be produced for biomass growth, biomass cannot be produced in the absence of nutrients. We emphasize that we evaluated the pan-metabolic network’s viability on specific carbon sources and its ability to produce secondary carbon sources without any quantitative evaluation of fluxes, for which spurious cycles might be a problem.

### Random viable metabolic networks

We wanted to study the niche construction capacity not only of *E*. *coli*, but of multiple networks of similar complexity that do not share *E*. *coli*’s or any other organism’s evolutionary history. Any one metabolic network can be thought of as a subset of reactions drawn from the set of all metabolic reactions feasible in a living organism, i.e., the pan-metabolic network. In the enormous space of all possible metabolic networks only a tiny fraction is viable on any one carbon source, i.e., they can produce biomass when this carbon source is the sole carbon source. We focused on such viable networks, and to sample them from the space of such networks, we used a technique based on Markov Chain Monte Carlo (MCMC) sampling [56,57], which samples networks during long random walks in the space of all metabolic networks of a given size. The statistical theory behind MCMC sampling [96] shows that its random walks are ergodic, i.e., roughly speaking, they are equally likely to visit all metabolic networks in a connected region of the space of such networks. In previous work, we have shown that in the space of all possible networks, networks viable on a specific carbon source form indeed a subset connected by single reaction changes [97]. One requirement of the method is that random walks have a sufficiently long burn-in period to ensure that any “memory” of the starting network of such a random walk has decayed. In previous work [56], we determined that a burn-in period of 5000 reaction changes is sufficient for this purpose. When this requirement is met, the method essentially ensures that the sampled networks contain a random complement of reactions, with no similarity to the starting network in excess of that required for viability on a specific carbon source.

The method starts from an initial network, which we chose as a network that is viable on glucose as a sole source of carbon and that has the same number of reactions (2583) as *E*. *coli*.(The computational cost of MCMC sampling prevented us from exploring other primary carbon sources.) To create this initial network, we first performed Flux Balance Analysis on the pan-metabolic network, with glucose as the only source of carbon. Of all reactions in the pan-metabolic network, 1263 reactions showed non-zero flux and were included in the initial network, which ensured viability on glucose. We chose the remaining (1320) reactions needed to arrive at an equal number of reactions as *E*. *coli* at random from the pan-metabolic network. From this initial network the MCMC method creates a long sequence of modified networks. In our implementation of the method, a new network is created by a reaction swap, in which a reaction from the existing network is randomly chosen for deletion, while a randomly chosen reaction (that is not yet present in the network) from the pan-metabolic network is added to the network. If the network remains viable after this reaction swap, the swap is accepted, and the network is modified with a further reaction swap. In contrast, if the reaction swap disrupts viability on glucose, the swap is rejected and a new swap is tried. Modifying metabolic networks through reaction swaps ensures that the number of reactions in the network remains constant (and equal to the number of reactions in the initial network). As the number of reaction swaps increases, the number of reactions that the altered networks share with the initial network becomes smaller and smaller, until the complement of reactions has become effectively randomized after 5000 successful swaps [95]. We stored such a randomized network (which is still viable on glucose) for further analysis after 5000 successful swaps.

We performed 500 independent such random walks, thus creating 500 metabolic networks all viable on glucose and containing as many reactions as the network of *E*. *coli* does. Each of them is the end point of a sequence of 5000 successful reaction swaps. This procedure is very time consuming, and to accelerate it, we first determined the reactions that are essential for growth on glucose in the pan-metabolic network, and did not subject these (169) reactions to deletions.

We note that we did not alter the exchange reactions of the starting network, which ensures that in the randomized networks the same metabolites can be exchanged with the environment as in *E*. *coli*. We also note that random networks contain a large number of reactions (1197±36) that cannot carry non-zero flux in any of the environments we consider, i.e., they are unconditionally blocked. Even though all networks analyzed in this work contain unconditionally blocked reactions, the numbers observed for the random viable networks are especially large.

Because we created random networks by sampling reactions from the pan-metabolic network, these networks may also contain spurious cycles [75–77]. For this reason we analyzed them similarly to the pan-metabolic network, evaluating only viability on specific carbon sources and the ability to produce secondary carbon sources, without any quantitative evaluation of fluxes which are most affected by spurious cycles.

We implemented our sampling procedure in python, using the cobrapy package [98] to perform flux balance analysis to check for viability on glucose of the networks created after each reaction swap.

## Supporting information

S1 Text Nutrient availability limit calculation. (DOCX)

S2 Text Biomass yield, maximal production and cost. (DOCX)

S3 Text The limits of coexistence when strains compete for the primary carbon source. (DOCX)

S4 Text Analytical analysis of the limits for coexistence. (DOCX)

S1 File Secondary carbon sources on glucose. The table contains the names, biomass yield, maximal production rate, and cost of each of the 58 secondary carbon sources that *E*. *coli* can produce when growing on a glucose minimal medium.
(ODS)

S2 File Pan-metabolic network in sbml format. (XML)

S1 Dataset 500 random networks viable on glucose. The folder contains 500 files, each corresponding to a random network obtained sampling (Methods, section Pan-metabolic and random networks) from the pan-metabolic network. Each network is represented with a sequence of zeros and ones where one and zero indicates presence or absence of the reaction in the random network respectively.
(ZIP)

S1 Fig Dynamics in the chemostat for various initial conditions. Horizontal axes in all panels indicate time in hours. Panels (A) and (B) show the biomass values of P and C respectively (vertical axes), as a function of time, while changing the initial biomass ratio of P and C (see color legends in both panels). Panels (C) and (D) show the biomass values of P and C respectively, as a function of time when changing the total initial biomass, while maintaining the ratio of P to C biomass at a constant value of 0.5 (see color legends in both panels). Panels (E) and (F) show the biomass values of P and C, respectively, as a function of time for two scenarios. In the first, the chemostat is initiated with equal amounts of P and C biomass at time zero (red line). In the second, the chemostat is initiated just with P, and C is introduced at various times in an amount equal to that of P at time zero (see color legend).The chemostat composition and all other parameters used in these simulations are identical to those described in the main text (Section “Simulating chemostat dynamics with dynamic FBA” in methods).
(PDF)

S2 Fig **Steady state biomass of P (left panel) and of C (right panel) in the chemostat**. The amounts of biomass are shown as a function of P’s acetate production rate (vertical axis) and of C’s glucose consumption rate (horizontal axis), which are expressed as percentages of the maximal acetate production rates and glucose consumption rates that permit coexistence of metabolically distinguishable strains P and C. Note the nonlinear scale used at low values of *p*_*ac*,*P*_ (vertical axis). The amount of biomass is indicated by a color gradient (see color legend) in the region where the two strains can coexist. Note that for parameter combinations where no acetate is produced and where glucose consumption is higher than 99% of *c*_*glc*,*C*_, both strains coexist but are metabolically indistinguishable, because both completely respire glucose to carbon dioxide. Areas (parameter combinations) where coexistence is not possible are shown in light grey (only P persists), dark grey (only C persists) and black (neither strain persists).
(PDF)

S3 Fig Steady state glucose consumption ratio. The figure shows the ratio of glucose consumption rates of strains C and P, as a function of P’s acetate production rate (vertical axis) and of C’s glucose consumption rate (horizontal axis), expressed as percentages of the maximal acetate production rates *p*_*ac*,*P*_ and glucose consumption rates *c*_*glc*,*C*_ that permit coexistence of metabolically distinguishable strains P and C. As in S3 Fig, depending on the values of *c*_*glc*,*C*_ and *p*_*ac*,*P*_, the chemostat in steady state may either contain no biomass (black region), only strain P (light grey), only strain C (dark grey), or both P and C (color). Colors from blue to yellow (see color bar) indicate the ratio of C’s glucose consumption rate and P’s glucose consumption rate when both strains are present in the chemostat, which varies between zero (when C does not consume glucose) and one (when C and P consume glucose at the same rate). The glucose consumption ratio is large (green, orange and yellow) when the producer strain produces little acetate (small *p*_*ac*,*P*_) while the consumer strain C consumes a lot of glucose (high *c*_*glc*,*C*_). The maximally possible glucose consumption ratio of one (bright yellow) is observed only when both strains are metabolically indistinguishable, i.e., when P produces no acetate and C uses glucose as the only source of carbon. The lower panel magnifies part of the upper panel for low values of acetate production (*p*_*ac*,*P*_ < 30) to appreciate how glucose consumption changes at these values.
(PDF)

S4 Fig Correlation between maximal production rate and cost. The figure shows the maximal production rate and cost for each of 58 secondary carbon source (grey circles) that *E*. *coli* can produce when growing on glucose. The black arrow indicates the data for acetate. See S2 Text for a description of how these quantities are calculated.
(PDF)

S5 FigSteady state biomass ratios for different secondary carbon sources.The figure is a different representation of the data shown in Fig 2D. The vertical axis shows the steady state biomass ratio C/P as a function of the product (horizontal axis) of maximal production ($p_{m}^{max}$) and biomass yield (*α*_*m*_) of the secondary carbon sources considered here (horizontal axis). Each circle in the plot corresponds to a secondary carbon source that *E*. *coli* can produce when glucose is the primary carbon source. Circle colors indicate the product of maximal production ($p_{m}^{max}$) and biomass yield (*α*_*m*_) of a secondary carbon source. This product equals 1.26 h^-1^ for acetate. The biomass ratio is shown for 1, 10 and 30% of the maximally possible secondary carbon source production flux, as indicated by the numbers in the panel.(PDF)

S6 FigSteady state biomass of all cross-feeding strain pairs, when glucose is the primary carbon sources and when the consumer strain consumes different amounts of glucose.Steady state biomass of producer strain P (vertical axes) and consumer strain C (horizontal axes) at different percentages of the maximum synthesis rate ($p_{m}^{max}$), i.e., the synthesis rate of the secondary carbon source beyond which the producer strain P is flushed out of the chemostat, for each of 54 secondary carbon sources (grey lines, one line per carbon source). Circles are placed at 1, 5, 10–90, 95 and 99% of the maximum synthesis rate. The dashed-dotted line indicates the maximally achievable total steady-state biomass (0.78 gDW/l), which is obtained when glucose is metabolized completely to CO_2_ by the producer strain, without synthesis of any secondary carbon source. The dashed line indicates where both strains have identical biomass. Circle colors indicate the product of maximal production ($p_{m}^{max}$) and biomass yield (*α*_*m*_) of a secondary carbon source. This product equals 1.26 h^-1^ for acetate (black line superposed with blue circles).The consumer strain has a consumption rate of glucose *c*_*glc*,*C*_ that is equal to 0% (A), 54% (B), and 99% (C) of the rate it needs to persist on glucose alone in the chemostat. Panel A is identical to Fig 2D and merely shown to facilitate comparison.(PDF)

S7 Fig Carbon sources and associated statistics for *E*. *coli*. The outermost circle lists all of *E*. *coli*'s carbon sources (as in Fig 2A), ordered clockwise according to biomass yield, starting from formate (for, 9’ o’clock). Green circles (solid green scale bar from center to top right) indicate the biomass yield of each carbon source. Black circles (solid black scale bar from center to top left) indicate the number of carbon atoms of the carbon source. Blue circles (solid blue scale bar from center to lower left) indicate the number of secondary carbon sources that can be produced when *E*. *coli* grows on a given primary carbon source. Red circles (solid red logarithmic scale bar from center to down right) indicate from how many primary carbon sources this carbon source can be produced as a secondary carbon source (if it can be produced at all). Most secondary carbon sources can be produced from all primary carbon sources (ln(179) = 5.2 on the red scale), but some can be produced only from few primary carbon sources. Among them is glucose (grey arrow), which can be produced only from four primary carbon sources.
(PDF)

S8 Fig Primary and secondary carbon sources in *E*. *coli* and in the pan-metabolic network. (A) Histogram of the number of secondary carbon sources that can be produced per primary carbon source in *E*. *coli* (blue dots in S7 Fig). (B) Histogram of the number of primary carbon sources from which each of the 83 secondary carbon sources in *E*. *coli* can be produced (see also red circles in S7 Fig). (C) and (D), like (A) and (B) but for the pan-metabolic network.
(PDF)

S9 Fig Chemostat dynamics of a glucose consumer-acetate producer community for different transport-associated parameters. We model transport limitation with Michaelis-Menten kinetics with parameters *V*_*max*_ and *k*_*M*_ (Methods). Horizontal axes in all panels indicate time in hours. Panels (A) and (B) show the biomass values of P and C, respectively (vertical axes), as a function of time, while changing *k*_*M*_ (expressed in mM, see color legends in both panels). The range of *k*_*M*_ values simulated covers 60% of all *k*_*M*_ values present in Brenda database [99] (The median *k*_*M*_ in the database is approximately 0.1 mM, with 60% of *k*_*M*_ values between 0.001 and 1 mM [89]).Panels (C) and (D) show the biomass values of P and C, respectively (vertical axes), as a function of time, while changing *V*_*max*_ (in mmol gDW^-1^ h^-1^,see color legends in both panels). If, for any one consumed metabolite, *V*_*max*_ is not high enough to permit growth at the dilution rate, the population will go extinct. Values of *V*_*max*_ above this minimalvalue that permits growth at the dilution rate can alter the transient biomass dynamics but does not affect the steady state biomass. The chemostat composition and all other parameters used in these simulations are identical to those described in the main text (Section “Simulating chemostat dynamics with dynamic FBA” in methods). We ended any one simulation when a population had reached steady state, which is the reason why the lines end at different time points.
(PDF)

S10 Fig Hypothetical example to illustrate the concepts of primary and secondary carbon source and the relationship between pan-metabolic network and random viable networks. Panel (A) shows a hypothetical pan-metabolic network comprising 10 internal and 5 transport reactions. Panel (B) shows four networks created by randomly selecting 4 internal reactions (in black) from the pan-metabolic network. In this hypothetical example, ATP must be produced from at least one of the environmental nutrients (A, D, F and G) for a network to be viable. All four networks are viable on A if nutrient H is available in the environment. The random networks 2, 3 and 4 are also viable on metabolites D, F and F and G respectively. If a metabolite on which a network is viable can be produced when nutrient A is consumed, the metabolite is a secondary carbon source (green). (C) Histogram of the number of secondary carbon sources per random network, for the four random networks considered here. (D) Rank plot of secondary carbon sources (horizontal axis) that can be produced by at least one random network when A is used as a primary carbon source, ranked by the fraction of random viable networks (vertical axis) by which the secondary carbon source can be produced.
(PDF)
