# Supplementary material for: An enormous potential for niche construction through bacterial cross-feeding in a homogeneous environment
Source: PLoS Comput Biol. 2018 Jul 24;14(7):e1006340. doi: 10.1371/journal.pcbi.1006340 (PMC6080805; doi:10.1371/journal.pcbi.1006340)
Supplement: S1 Text — (DOCX) [file pcbi.1006340.s001.docx]

**Calculating the nutrient availability limit**

In this section, we describe how to determine the nutrient availability limit for each environmental nutrient, and for each strain in our simulated chemostat. This maximal availability is required to calculate the maximum nutrient uptake rate, which FBA requires to determine the maximal biomass production rate.

The two strains in our chemostat do not necessarily compete for any one nutrient. For example, a nutrient may be available in sufficiently high concentrations that it does not limit growth, or only one of the strains may be able to use the nutrient for growth. In such cases it is straightforward to determine the nutrient availability limit: It is calculated by dividing the total concentration of the nutrient by the biomass of the strains consuming the nutrient.

In contrast, it is less straightforward to determine a nutrient’s availability limit when the strains compete for this nutrient. Some authors [1] select randomly to which strain they allocate the whole amount of nutrient existing in the environment at a given time. The remaining nutrients can be consumed by the other strain. This strategy is reasonable but it may alter the ecological dynamics of the strains depending on which strain is selected for nutrient allocation at each time step of the simulation. Other authors [2] divide the nutrient’s concentration by the total biomass (adding the biomass of all the strains) and allocate equal amounts of nutrient to be consumed by each strain. Although this approach is also reasonable, it eliminates frequency dependency effects that can arise when a strain with small biomass consumes a nutrient that is not consumed by the other strain. Specifically, consider two strains P and C that are present in the chemostat. Strain P is abundant whereas C is rare. Both can in principle consume the nutrient *m* (they express the necessary enzymes to do so), but when strain P grows its biomass at the maximally possible rate, as predicted by FBA, it does not consume *m*, perhaps because maximal biomass growth is achieved with other available nutrients. If we calculated the nutrient availability limit for C by simply dividing the concentration of *m* by the sum of the biomass values of P and C, we would allow little of *m* to be consumed by C. In consequence, C’s growth rate would remain low, and most of *m* would remain unutilized in the chemostat, because it is not consumed by the much more abundant strain P.

To overcome undesirable artifacts like this, we pursued the following, third strategy, which is more appropriate for our purpose. At each time step$t_{i}$, both strains are allowed to consume a given nutrient, but we make the amount allocated to each strain dependent on their consumption at the previous time step$t_{i-1}.$By doing so, we encourage continuity with respect to the strains’ nutrient consumption. With two strains (P and C), there are four possibilities regarding nutrient consumption at time$t_{i-1}$: 1) both strains consumed the nutrient, 2) only strain P did, 3) only strain C did, 4) none of the strains did. In case 1) both strains consumed the nutrient at time $t_{i-1}$, so we simply calculate the nutrient availability limit by dividing the nutrient's concentration by the total biomass. In case 2), where only strain P consumed the nutrient, we calculate the nutrient availability limit for P by dividing the metabolite's concentration by the biomass of P. At time step $t_{i}$, since the environment has changed with respect to time $t_{i-1}$ it may have become beneficial for C to consume the nutrient of interest, i.e., doing so may help increase the growth rate of C. To allow for this possibility, we calculate the nutrient availability limit for C by dividing the nutrient's concentration by the total biomass of both strains. For case 3) the nutrient availability limitcalculates analogously to case 2, but with strain identities reversed. In the last scenario 4), where none of the strains had consumed the nutrient at the previous time step, we permit both strains to consume the available nutrient. In this case, the nutrient availability limit for each strain is obtained by dividing the nutrient concentration by the strain’s biomass. In theory, under scenario 2, 3 or 4, either strain may consume all nutrient at time $t_{i}$, and total nutrient consumption may thus exceed the amount of available nutrient. In practice, this will not occur if the time steps are small enough, and for our time step size (0.1h^-1^) it never occurred. Note that in scenario 2, 3 or 4, the availability limit of the nutrient may differ between the strains.

Overall, this strategy provides continuity in the nutrient consumption pattern of the two strains from one time step to the next, while still permitting change in this pattern. We can formalize the calculation of the nutrient availability limit as follows. For strain P at time $t_{i}$the availability limit of a nutrient (metabolite) with concentration *M* calculates as

$M/((X_{P}+f_{M,C}(t_{i-1}){\times X}_{C})\times\Delta t)$ (1)

Here, $f_{M,C}\left( t_{i-1} \right)$can take values of one and zero, corresponding to the situation where strain C consumed or did not consume the nutrient at time $t_{i-1}$, respectively. The variables $X_{P}$ and $X_{C}$denote the biomass values of strains P and C, respectively. With analogous notation, the availability limit of this nutrient for strain C at time $t_{i}$ calculates as

$M/((X_{C}+f_{M,P}(t_{i-1}){\times X}_{P})\times\Delta t)$ (2)

Literature Cited

1. Harcombe WR, Riehl WJ, Dukovski I, Granger BR, Betts A, Lang AH, et al. Metabolic resource allocation in individual microbes determines ecosystem interactions and spatial dynamics. Cell Reports. 2014; doi:10.1016/j.celrep.2014.03.070

2. Chiu HC, Levy R, Borenstein E. Energent biosynthetic capacity in simple microbial communities. PLOS computational biology. 2014;
