# Supplementary material for: An enormous potential for niche construction through bacterial cross-feeding in a homogeneous environment
Source: PLoS Comput Biol. 2018 Jul 24;14(7):e1006340. doi: 10.1371/journal.pcbi.1006340 (PMC6080805; doi:10.1371/journal.pcbi.1006340)
Supplement: S2 Text — (DOCX) [file pcbi.1006340.s002.docx]

**Biomass yield, maximal production and cost**

We define the biomass yield $\alpha_{m}$of a nutrient or metabolite as the growth rate a metabolism can achieve per consumed unit of flux of the metabolite. To compute this yield in *E. coli* for any one metabolite, we allowed an uptake rate ($J_{m}^{in}$) of 10mmol gDW^-1^ h^-1^of the metabolite, and maximized biomass production with Flux Balance Analysis. We then reported the yield as the maximal growth rate obtained per unit flux of metabolite consumed.

A second quantity relevant to our analysis is the maximal production rate of a metabolite$p_{m}^{max}$. We define this rate as the maximal rate of a metabolite’s production that allows the organism producing the metabolite to grow at a rate that is identical to the chemostat’s dilution rate *D*. In other words, it is the highest metabolite production rate at which the organism will not get flushed out of the chemostat over time. For the calculation of this rate, we performed FBA to maximize the production of the desired metabolite, while constraining the growth rate to the value of the dilution rate D.

A third relevant quantity is the metabolic cost of producing a metabolite. To calculate this cost, we computed the maximal biomass growth rate with FBA twice, first without any additional constraints, and then by requiring that the focal metabolite is produced at a rate of 1 mmol gDW^-1^ h^-1^. We quantify the cost of producing the metabolite as the reduction in growth rate when the metabolite is produced at this rate.

We note that the cost and maximal production rate of a metabolite are closely related and show an inverse relationship. The greater the cost, the smaller the maximum rate that a metabolite can be produced at a given biomass growth rate (supplementary Fig S2).
