# Supplementary material for: An enormous potential for niche construction through bacterial cross-feeding in a homogeneous environment
Source: PLoS Comput Biol. 2018 Jul 24;14(7):e1006340. doi: 10.1371/journal.pcbi.1006340 (PMC6080805; doi:10.1371/journal.pcbi.1006340)
Supplement: S3 Text — (DOCX) [file pcbi.1006340.s003.docx]

**The limits of coexistence when strains compete for the primary carbon source**

In the main text we explored a simple scenario in which the strain C consuming a secondary carbon source cannot metabolize the primary carbon source ($c_{glc,C}=0$). This scenario leads to coexistence of strains P and C. In this section we explore the consequences of a more realistic but complex scenario that emerges when strain C can consume not only the secondary carbon source, but also the primary carbon source (Fig 1A of main text with $c_{glc,C}>0$). In this scenario, the strains compete for the primary carbon source. To identify the conditions for their coexistence, we varied the producer’s acetate synthesis rate between 0 and 100% of the maximally possible value $p_{ac,P}^{max}$beyond which the strain would eventually be flushed out of the chemostat. To model the consumer strain’s dynamics, we first used FBA to determine the minimal glucose consumption rate $c_{glc,C}^{min}$ C would need to persist in the chemostat by itself (in the absence of the other strain), if glucose were the only carbon source. This minimal glucose consumption rate is 2.04mmol gDW^-1^ h^-1^ for our dilution rate of D=0.02h^-1^. We then varied C’s glucose consumption rate $c_{glc,C}$ between zero and a value larger than this minimum, at which C can persist in the chemostat by consuming only glucose.

Fig S2 shows the steady-state composition of the chemostat for different values of glucose consumption by C (horizontal axis), expressed as a percentage of the minimal glucose consumption rate $c_{glc,C}^{min}$ under which strain C can persist on glucose alone, and acetate production by P (vertical axis),expressed as a percentage of the maximum acetate production rate $p_{ac,P}^{max}$ under which strain P persists. Depending on these rates, C alone may persist (dark grey), P alone may persist (light grey), neither strain may persist (black), or both strains may persist (coexistence, colored).

When C’s glucose consumption exceeds$c_{glc,C}^{min}$, such that it could persist on glucose alone, then only two persistence outcomes are possible. First, only C may persist (Fig S2, dark grey). This occurs if P produces acetate, which inflicts a metabolic cost on P. This cost reduces P’s growth rate, and thus causes a growth disadvantage relative to C, which leads to P’s eventual extinction. Second, the two strains coexist but are metabolically indistinguishable (Fig S2 bright yellow). This occurs when P produces no acetate, such that C uses the same resources as P for growth (Fig S3). Since the two strains harbor identical metabolic networks, they metabolize glucose in the same way, respiring it completely to carbon dioxide, and are thus metabolically indistinguishable.

When C’s glucose consumption is insufficient for its persistence ($c_{glc,C}<c_{glc,C}^{min}$) three outcomes are possible. First, if P produces no acetate, P persists but C goes extinct (Fig S2, light grey), because C consumes insufficient glucose for its persistence and because no acetate is available to supplement its glucose consumption. Second, if P produces acetate at the maximal rate $p_{ac,P}^{max}$, P and C go extinct. P goes extinct because it has to produce more acetate than it is capable of while persisting. After P’s extinction, the acetate it produced will eventually disappear from the chemostat, which leads to the extinction of C, because C cannot persist on glucose alone. For intermediate acetate production (0<$p_{ac,P}$<$p_{ac,P}^{max}$) the two strains coexist stably and cross-feed.

In the experiments that motivated this work [1] the steady state biomass of the producer strain was reported to be approximately nine times that of the consumer strain. We predict this ratio when P produces little acetate (≈1% of the maximal acetate production rate $p_{ac,P}^{max}$) and C consumes glucose at a rate of up to ≈70% of the minimum rate $c_{glc,C}^{min}$needed for growth on glucose alone. To our knowledge, the maximal acetate production$p_{ac,P}^{max}$ and minimal glucose consumption rates have not been measured experimentally. However, our prediction is reasonable given the short divergence times and high metabolic similarity between strains, because it requires only that the producer strain produces very little acetate and the consumer strain consumes high amounts of glucose.

Literature cited

1. Rosenzweig RF, Sharp RR, Treves DS, Adams J. Microbial evolution in a simple unstructured environment: genetic differentiation in Escherichia coli. Genetics. 1994;
