# Supplementary material for: An enormous potential for niche construction through bacterial cross-feeding in a homogeneous environment
Source: PLoS Comput Biol. 2018 Jul 24;14(7):e1006340. doi: 10.1371/journal.pcbi.1006340 (PMC6080805; doi:10.1371/journal.pcbi.1006340)
Supplement: S4 Text — (DOCX) [file pcbi.1006340.s004.docx]

**Analytical analysis of the limits for coexistence**

In supplementary Fig S2 and Fig S3 we observed that for some combination of acetate production by P ($p_{ac,P}$) and glucose consumption by C ($c_{glc,C}$), two metabolically distinguishable strains cannot coexist. In this section, we analyze the conditions for coexistence analytically to demonstrate the generality of our observations. In the following, we use notation specific to glucose and acetate, but we emphasize that our conclusions are independent of the primary and secondary carbon source considered.

At a metabolic steady state ($ss$), if P and C strains are to coexist, the growth rates of the producer strain P and the consumer strain C must be equal to the dilution rate of the chemostat. In mathematical terms (see also equations (1) and (2) in the main text):

$\mu_{P}^{ss}=\mu_{C}^{ss}=D$ (1)

The producer strain P consumes glucose at some rate$J_{glc,P}^{in}$. Part of this glucose flux is used for acetate production and the rest is used for growth. The amount used for acetate production will depend on $p_{ac,P}$, i.e., the acetate flux to be produced. It will also depend on the ‘cost’ of acetate production,$\beta_{glc}^{ac}$, which we define as the growth reduction observed per unit of acetate flux produced, when a strain grows on glucose. The growth rate for the producer strain can then be expressed as

$\mu_{P}=\alpha_{glc}(J_{glc,P}^{in}-\beta_{glc}^{ac}p_{ac,P})$  **(2)**

In other words, strain P consumes glucose at a rate $J_{glc,P}^{in}$, and part of the consumed glucose ($\beta_{glc}^{ac}p_{ac,P}$) is used for acetate production. The remainder ($J_{glc,P}^{in}-\beta_{glc}^{ac}p_{ac,P}$) is used for growth, and the growth rate can be calculated by taking into account the biomass yield of glucose ($\alpha_{glc}$) (Supplementary Text S2).

Similarly, the growth rate of the consumer strain depends on the amount of glucose and acetate consumed and their respective biomass yields ($\alpha_{glc}$ and $\alpha_{ac}$) in the following way:

$\mu_{C}=\alpha_{glc}J_{glc,C}^{in}$+$\alpha_{ac}J_{ac,C}^{in}$ (3)

In metabolic steady state, the dilution flux *D* must be equal to the biomass production for any strain persisting in the chemostat (equation (1)), which allows us to derive a simple condition for the persistence of consumer strain C in the presence of strain P. Specifically, by combining equation (1) and (3), we obtain $D=\alpha_{glc}J_{glc,C}^{in,ss}$+$\alpha_{ac}J_{ac,C}^{in,ss}$ and therefore $J_{glc,C}^{in,ss}=(D-\alpha_{ac}J_{ac,C}^{in,ss})/\alpha_{glc}$. By definition, $J_{ac,C}^{in,ss}>0$ must hold for any acetate consumer strain C, and since $\alpha_{ac}$ is always positive, we obtain

$J_{glc,C}^{in,ss}<D/\alpha_{glc}$ (4)

This expression indicates that as long as the glucose consumption rate of strain C is lower than the dilution rate divided by the biomass yield of glucose, the metabolically distinguishable strains P and C can coexist. Higher dilution rates D will permit higher glucose consumptions by C simply because to sustain growth at higher dilution rate, more carbon must be consumed (either as primary or secondary carbon source). The same reasoning explains why consuming a primary carbon source with lower biomass yield than glucose permits higher primary carbon source consumption by C for coexistence. That is, a reduced biomass yield of the primary carbon source leads to a lower growth rate per unit flux, such that a higher rate of consuming C becomes acceptable for coexistence. (We note that this entire analysis assumes that some acetate exists in the medium to be consumed by strain *C*, i.e., that $0\leq p_{ac,P}\leq p_{ac,P}^{max}$).

We have determined the biomass yield of glucose in the *E. coli* metabolism (iJO1366) as $\alpha_{glc}=0.098$. With a dilution rate of D=0.2 used throughout this paper, equation (4) gives a maximum glucose consumption rate by C of 2.04mmol gDW^-1^ h^-1^ at which two metabolically distinguishable producer and consumer strains can coexist. This analytically derived rate explains the change from coexistence to non-coexistence we observed in our simulations (Fig S2 and S3) as the glucose consumption rate of strain C $\left( c_{glc,C} \right)$ increases from $c_{glc,C}=2.02$ (99%) to $c_{glc,C}=2.04$ (100%).

In addition to these considerations, we can also combine equations (1), (2) and (4) to obtain a value for their relative rate of glucose consumption that the strains must fulfill for coexistence. Expressed as a ratio

$\frac{J_{glc,C}^{in,ss}}{J_{glc,P}^{in,ss}}<1-\frac{\beta_{glc}^{ac}p_{ac,P}}{J_{glc,P}^{in,ss}}$ (5)

The inequality shows that although P and C can both consume glucose, coexistence requires that their consumption rates are unequal. Specifically, C’s glucose consumption rate must be lower than P’s glucose consumption rate, and how much lower is given by the cost of acetate production and the amount of acetate $p_{ac,P}$ to be produced (Fig S3). We emphasize again that the above analyses, in particular equations (4) and (5), are not specific to glucose and acetate, but apply to any primary and secondary carbon source pair.
