# Supplementary figures and images for: An enormous potential for niche construction through bacterial cross-feeding in a homogeneous environment
Source: PLoS Comput Biol. 2018 Jul 24;14(7):e1006340. doi: 10.1371/journal.pcbi.1006340 (PMC6080805; doi:10.1371/journal.pcbi.1006340)

A

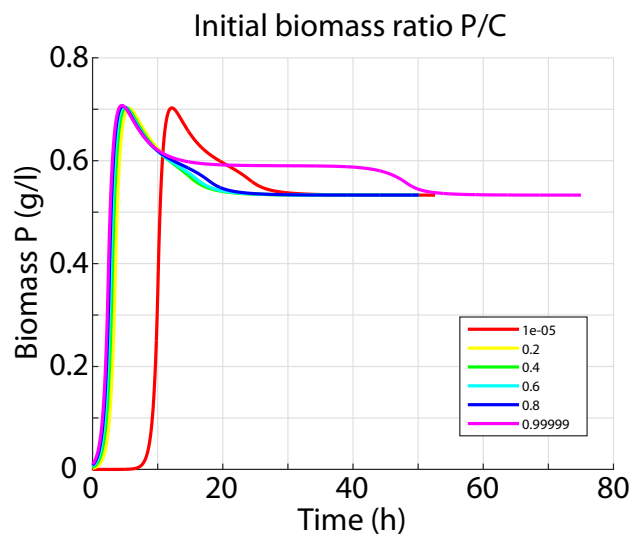

B

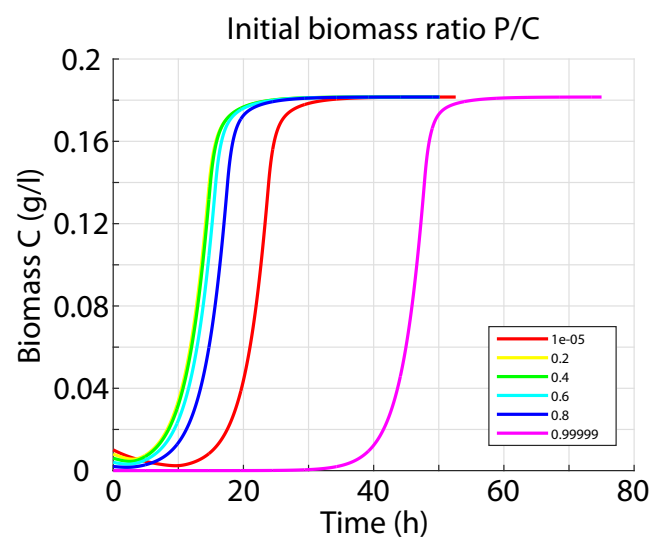

C

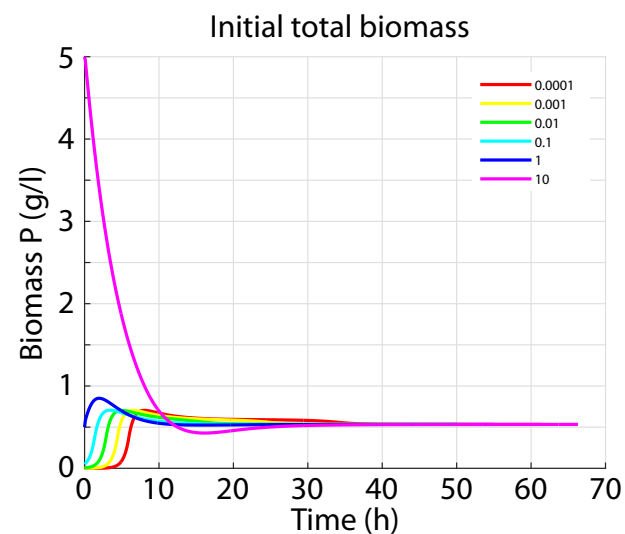

D

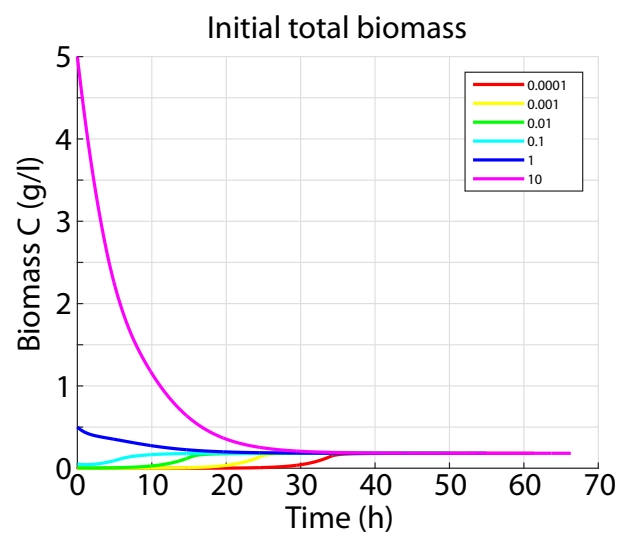

E

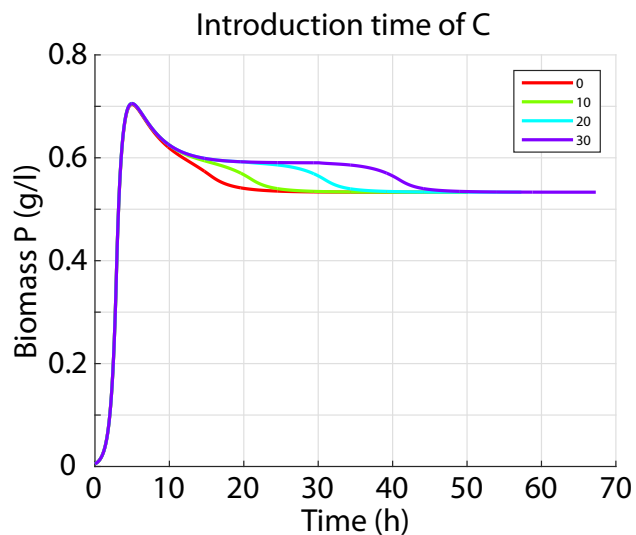

F

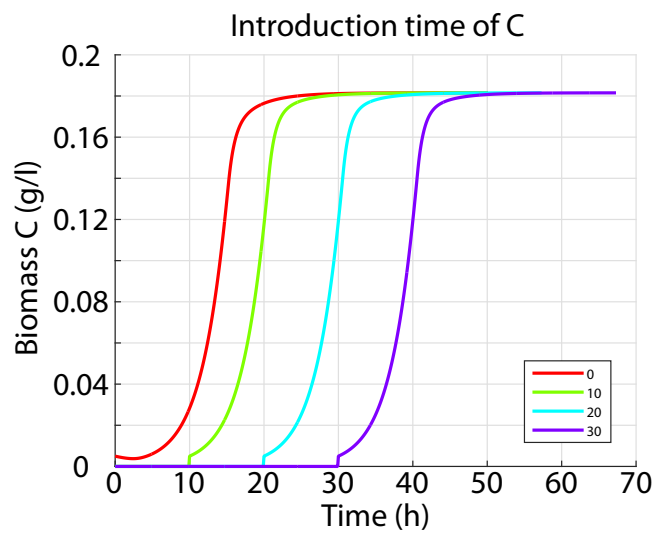

Supplement: S1 Fig — Horizontal axes in all panels indicate time in hours. Panels (A) and (B) show the biomass values of P and C respectively (vertical axes), as a function of time, while changing the initial biomass ratio of P and C (see color legends in both panels). Panels (C) and (D) show the biomass values of P and C respectively, as a function of time when changing the total initial biomass, while maintaining the ratio of P to C biomass at a constant value of 0.5 (see color legends in both panels). Panels (E) and (F) show the biomass values of P and C, respectively, as a function of time for two scenarios. In the first, the chemostat is initiated with equal amounts of P and C biomass at time zero (red line). In the second, the chemostat is initiated just with P, and C is introduced at various times in an amount equal to that of P at time zero (see color legend).The chemostat composition and all other parameters used in these simulations are identical to those described in the main text (Section “Simulating chemostat dynamics with dynamic FBA” in methods). (PDF) [file pcbi.1006340.s008.pdf]

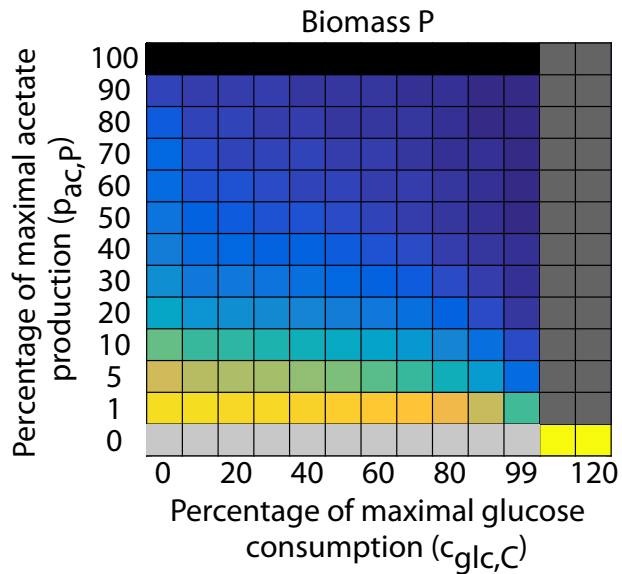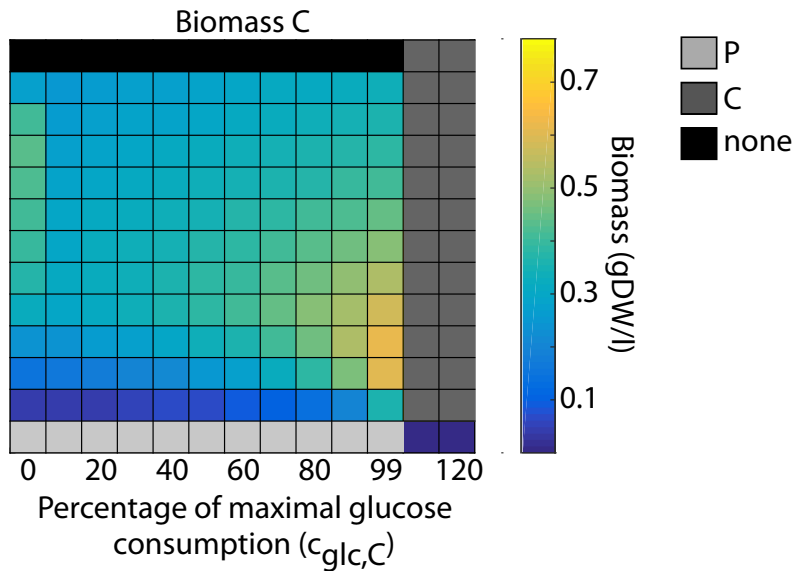

Supplement: S2 Fig — Steady state biomass of P (left panel) and of C (right panel) in the chemostat. The amounts of biomass are shown as a function of P’s acetate production rate (vertical axis) and of C’s glucose consumption rate (horizontal axis), which are expressed as percentages of the maximal acetate production rates and glucose consumption rates that permit coexistence of metabolically distinguishable strains P and C. Note the nonlinear scale used at low values of pac,P (vertical axis). The amount of biomass is indicated by a color gradient (see color legend) in the region where the two strains can coexist. Note that for parameter combinations where no acetate is produced and where glucose consumption is higher than 99% of cglc,C, both strains coexist but are metabolically indistinguishable, because both completely respire glucose to carbon dioxide. Areas (parameter combinations) where coexistence is not possible are shown in light grey (only P persists), dark grey (only C persists) and black (neither strain persists). (PDF) [file pcbi.1006340.s009.pdf]

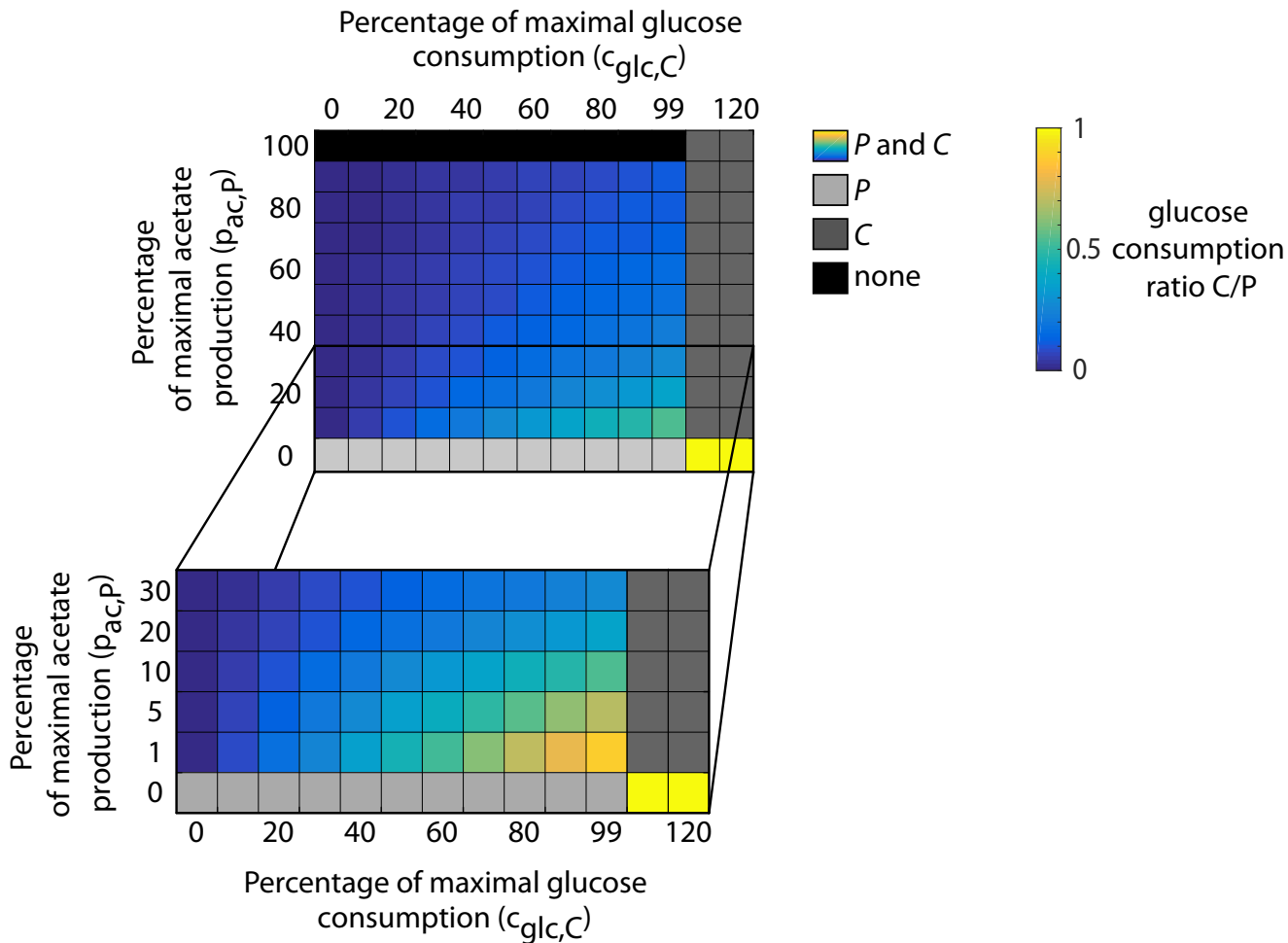

Supplement: S3 Fig — The figure shows the ratio of glucose consumption rates of strains C and P, as a function of P’s acetate production rate (vertical axis) and of C’s glucose consumption rate (horizontal axis), expressed as percentages of the maximal acetate production rates pac,P and glucose consumption rates cglc,C that permit coexistence of metabolically distinguishable strains P and C. As in S3 Fig, depending on the values of cglc,C and pac,P, the chemostat in steady state may either contain no biomass (black region), only strain P (light grey), only strain C (dark grey), or both P and C (color). Colors from blue to yellow (see color bar) indicate the ratio of C’s glucose consumption rate and P’s glucose consumption rate when both strains are present in the chemostat, which varies between zero (when C does not consume glucose) and one (when C and P consume glucose at the same rate). The glucose consumption ratio is large (green, orange and yellow) when the producer strain produces little acetate (small pac,P) while the consumer strain C consumes a lot of glucose (high cglc,C). The maximally possible glucose consumption ratio of one (bright yellow) is observed only when both strains are metabolically indistinguishable, i.e., when P produces no acetate and C uses glucose as the only source of carbon. The lower panel magnifies part of the upper panel for low values of acetate production (pac,P < 30) to appreciate how glucose consumption changes at these values. (PDF) [file pcbi.1006340.s010.pdf]

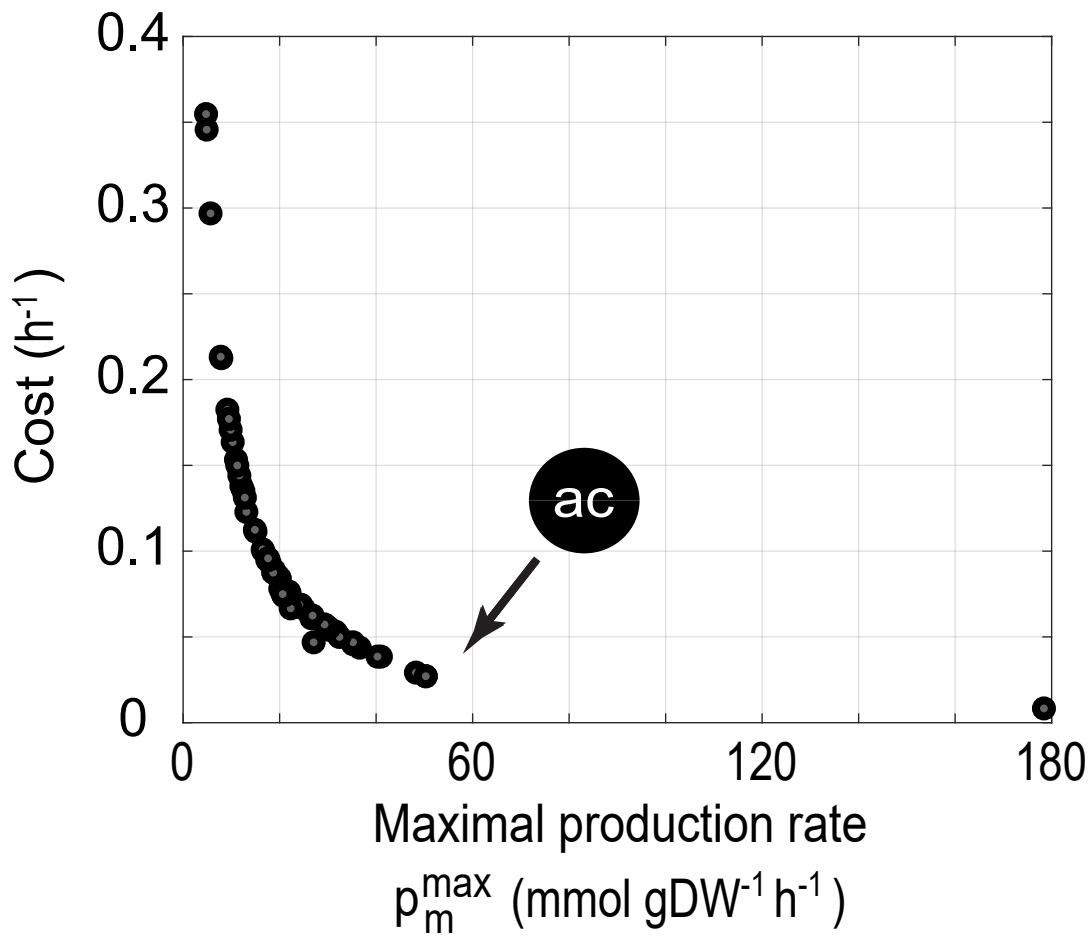

Supplement: S4 Fig — The figure shows the maximal production rate and cost for each of 58 secondary carbon source (grey circles) that E. coli can produce when growing on glucose. The black arrow indicates the data for acetate. See S2 Text for a description of how these quantities are calculated. (PDF) [file pcbi.1006340.s011.pdf]

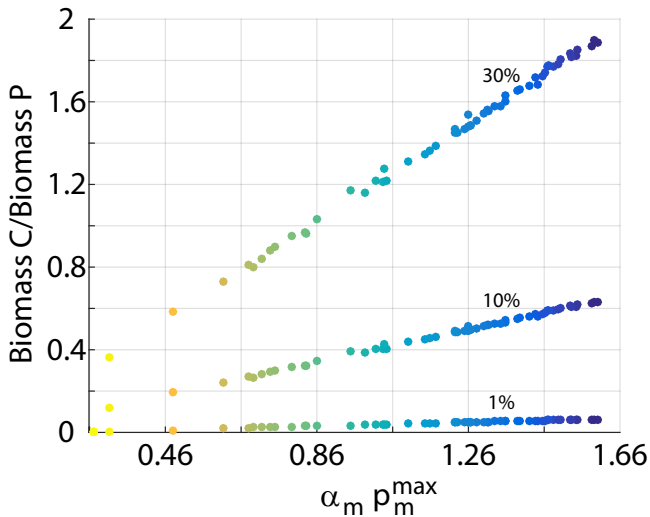

Supplement: S5 Fig — The figure is a different representation of the data shown in Fig 2D. The vertical axis shows the steady state biomass ratio C/P as a function of the product (horizontal axis) of maximal production (pmmax) and biomass yield (αm) of the secondary carbon sources considered here (horizontal axis). Each circle in the plot corresponds to a secondary carbon source that E. coli can produce when glucose is the primary carbon source. Circle colors indicate the product of maximal production (pmmax) and biomass yield (αm) of a secondary carbon source. This product equals 1.26 h-1 for acetate. The biomass ratio is shown for 1, 10 and 30% of the maximally possible secondary carbon source production flux, as indicated by the numbers in the panel. (PDF) [file pcbi.1006340.s012.pdf]

# Changing percentage of maximal glucose consumption ( $c_{glc,C}$ )

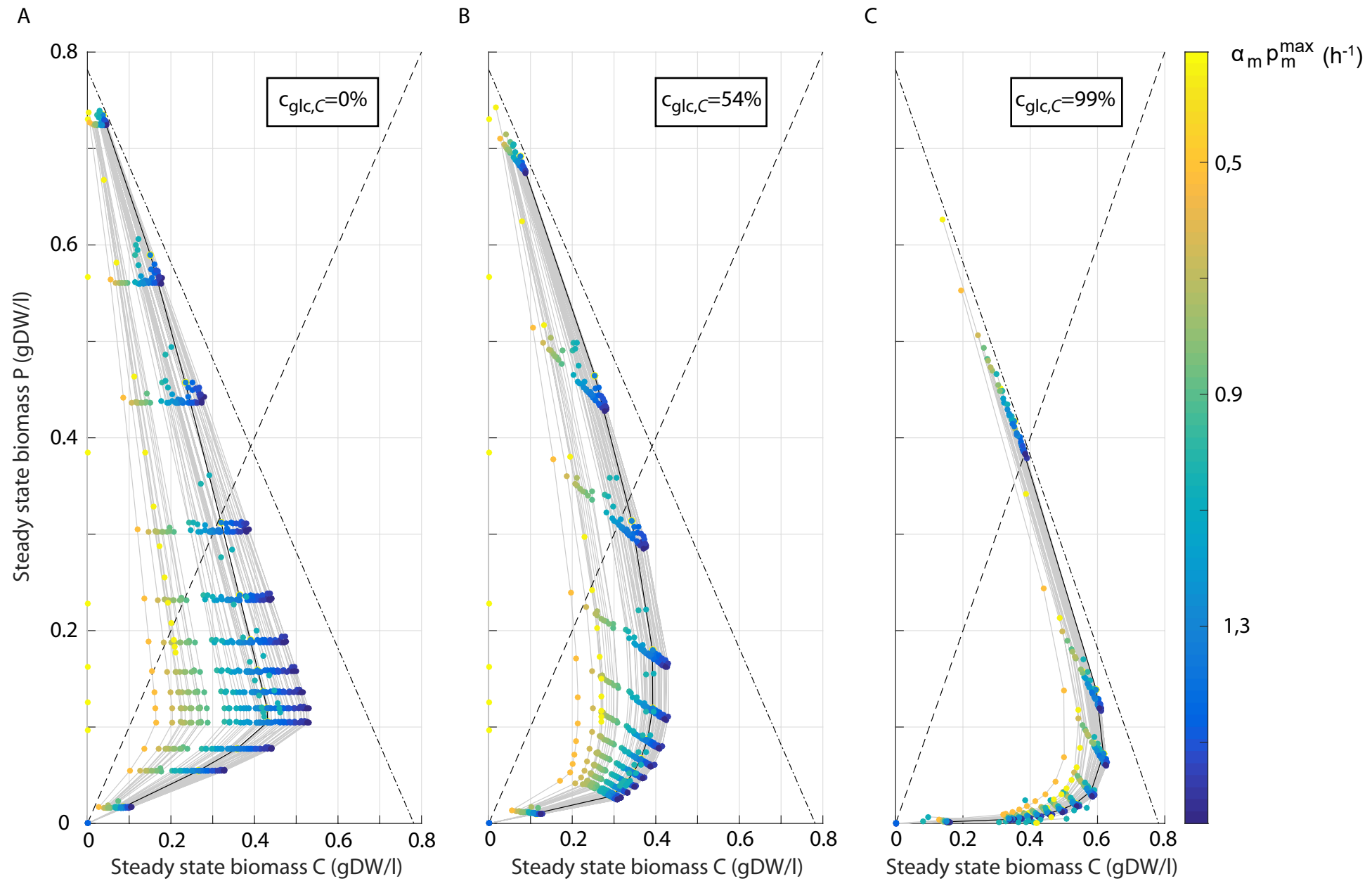

Supplement: S6 Fig — Steady state biomass of producer strain P (vertical axes) and consumer strain C (horizontal axes) at different percentages of the maximum synthesis rate (pmmax), i.e., the synthesis rate of the secondary carbon source beyond which the producer strain P is flushed out of the chemostat, for each of 54 secondary carbon sources (grey lines, one line per carbon source). Circles are placed at 1, 5, 10–90, 95 and 99% of the maximum synthesis rate. The dashed-dotted line indicates the maximally achievable total steady-state biomass (0.78 gDW/l), which is obtained when glucose is metabolized completely to CO2 by the producer strain, without synthesis of any secondary carbon source. The dashed line indicates where both strains have identical biomass. Circle colors indicate the product of maximal production (pmmax) and biomass yield (αm) of a secondary carbon source. This product equals 1.26 h-1 for acetate (black line superposed with blue circles).The consumer strain has a consumption rate of glucose cglc,C that is equal to 0% (A), 54% (B), and 99% (C) of the rate it needs to persist on glucose alone in the chemostat. Panel A is identical to Fig 2D and merely shown to facilitate comparison. (PDF) [file pcbi.1006340.s013.pdf]

A

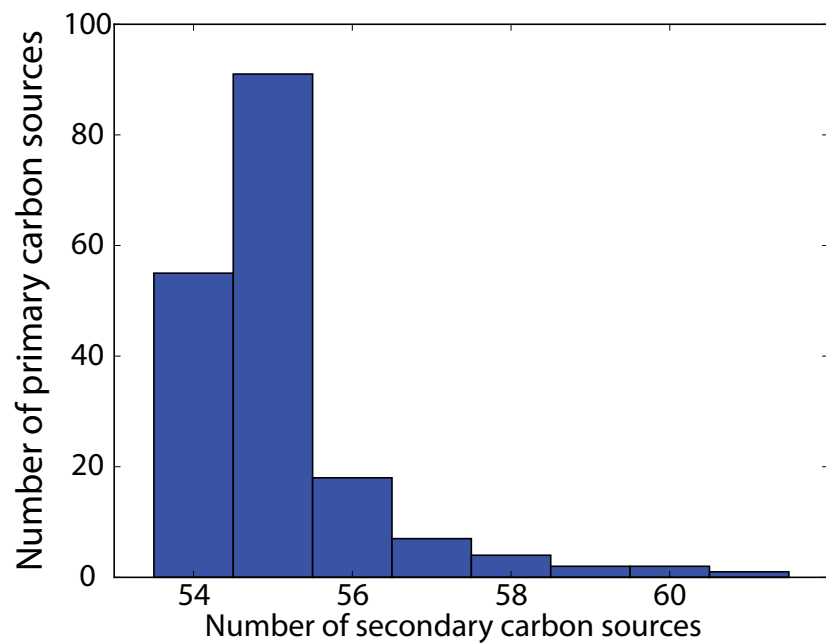

B

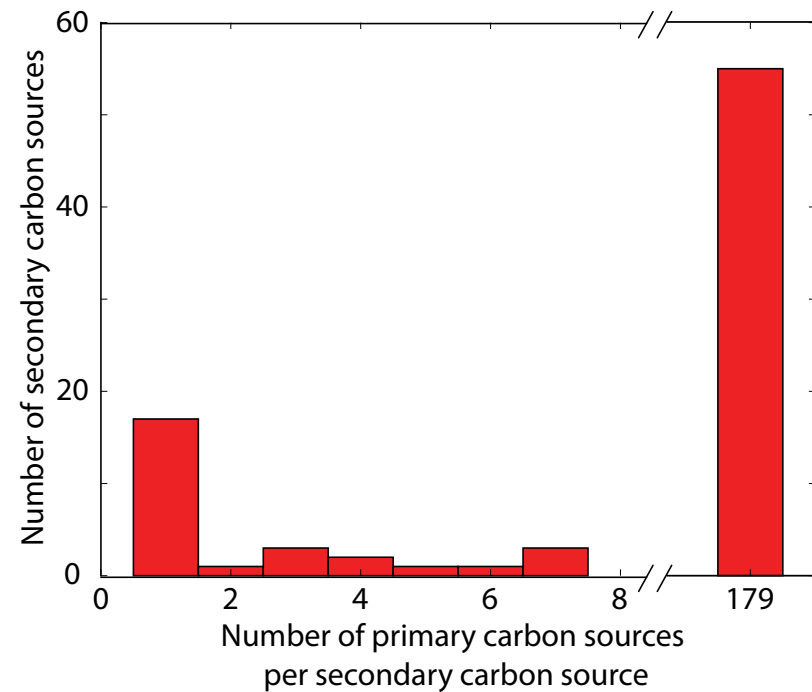

C

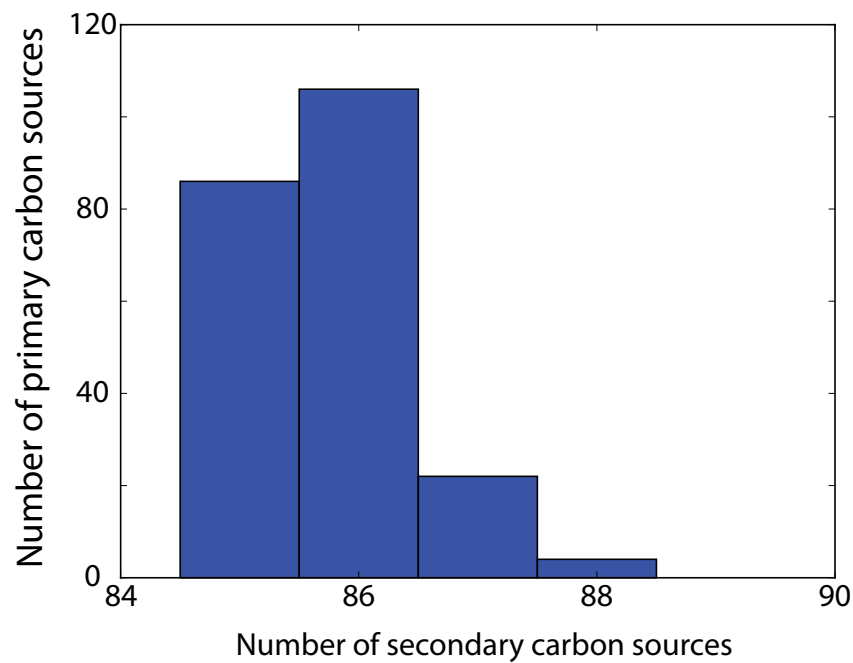

D

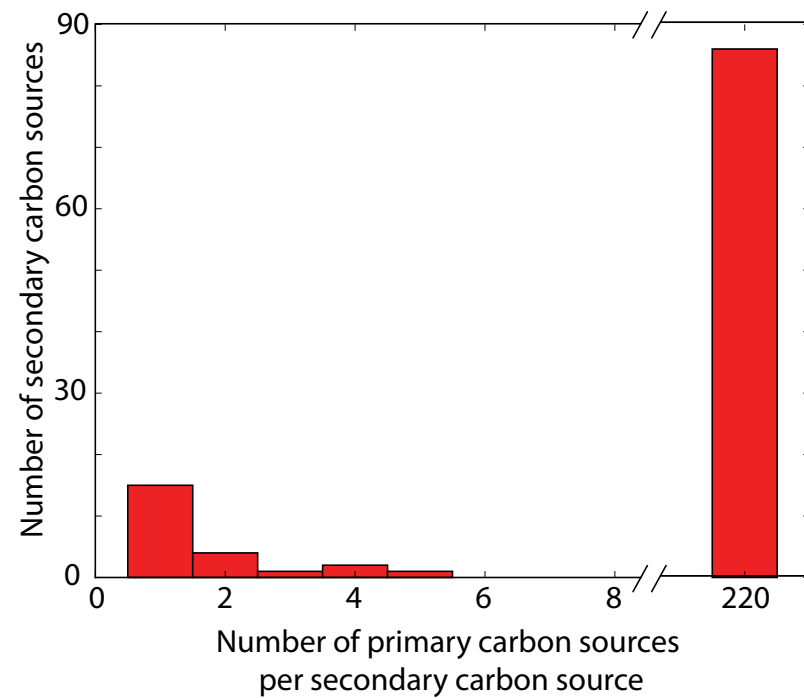

Supplement: S8 Fig — (A) Histogram of the number of secondary carbon sources that can be produced per primary carbon source in E. coli (blue dots in S7 Fig). (B) Histogram of the number of primary carbon sources from which each of the 83 secondary carbon sources in E. coli can be produced (see also red circles in S7 Fig). (C) and (D), like (A) and (B) but for the pan-metabolic network. (PDF) [file pcbi.1006340.s015.pdf]

A

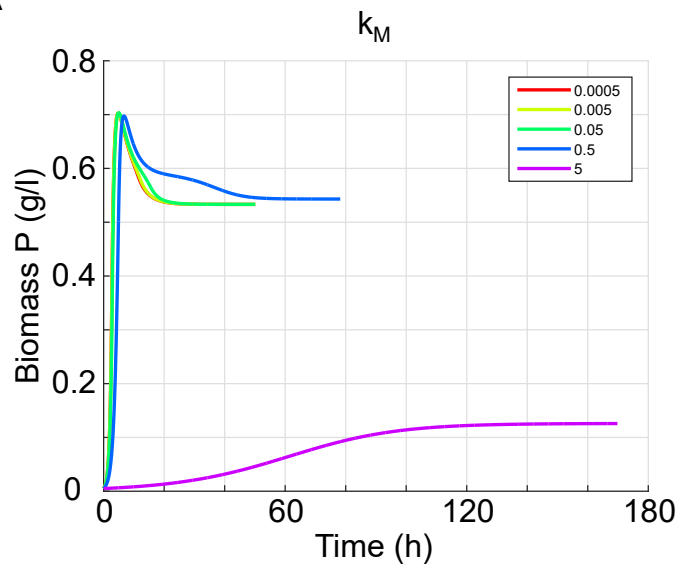

B

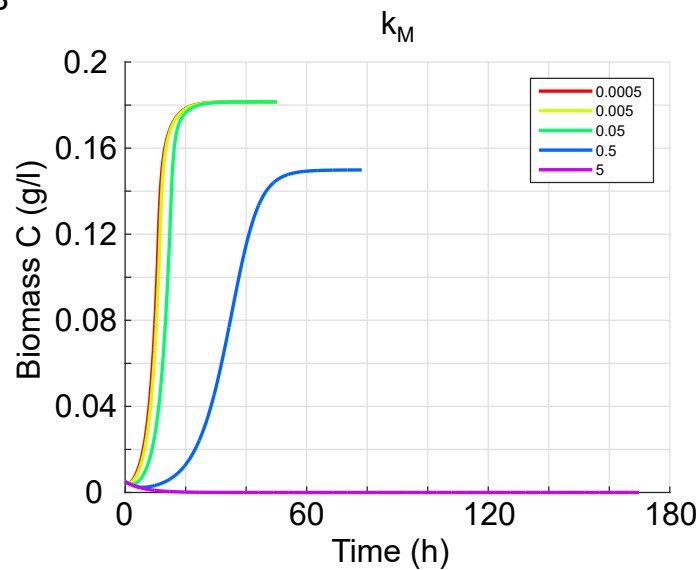

C

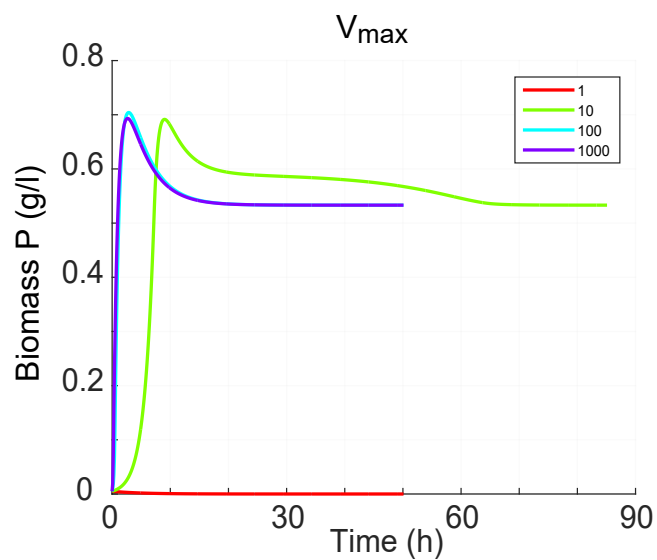

D

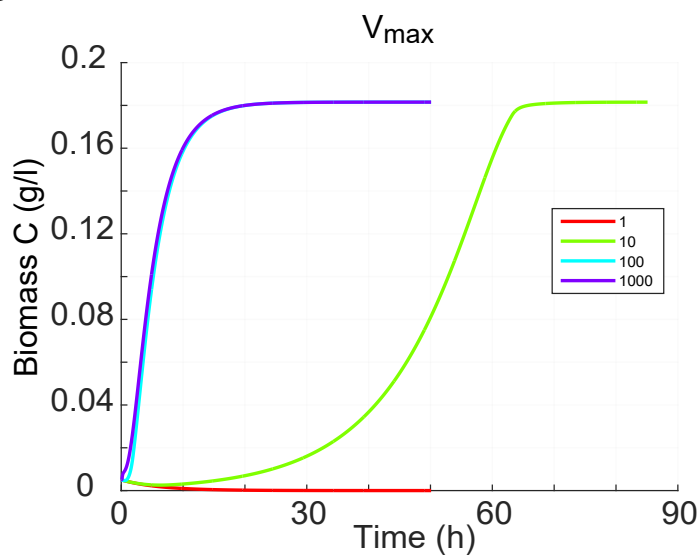

Supplement: S9 Fig — We model transport limitation with Michaelis-Menten kinetics with parameters Vmax and kM (Methods). Horizontal axes in all panels indicate time in hours. Panels (A) and (B) show the biomass values of P and C, respectively (vertical axes), as a function of time, while changing kM (expressed in mM, see color legends in both panels). The range of kM values simulated covers 60% of all kM values present in Brenda database [99] (The median kM in the database is approximately 0.1 mM, with 60% of kM values between 0.001 and 1 mM [89]).Panels (C) and (D) show the biomass values of P and C, respectively (vertical axes), as a function of time, while changing Vmax (in mmol gDW-1 h-1,see color legends in both panels). If, for any one consumed metabolite, Vmax is not high enough to permit growth at the dilution rate, the population will go extinct. Values of Vmax above this minimalvalue that permits growth at the dilution rate can alter the transient biomass dynamics but does not affect the steady state biomass. The chemostat composition and all other parameters used in these simulations are identical to those described in the main text (Section “Simulating chemostat dynamics with dynamic FBA” in methods). We ended any one simulation when a population had reached steady state, which is the reason why the lines end at different time points. (PDF) [file pcbi.1006340.s016.pdf]

A

Universal network

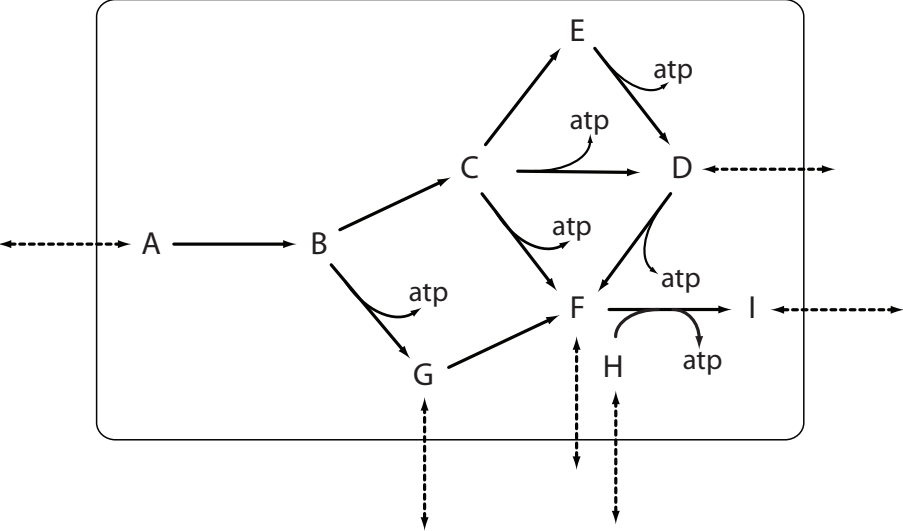

B

Random network 1

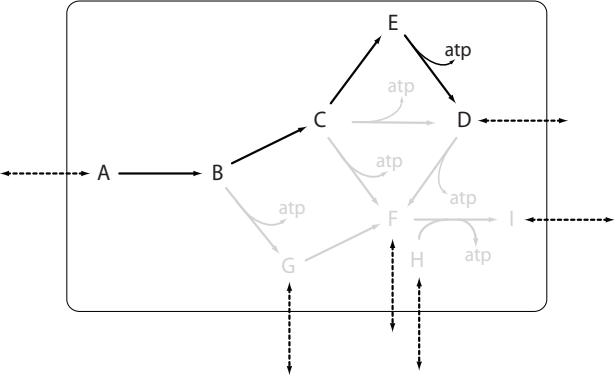

Random network 2

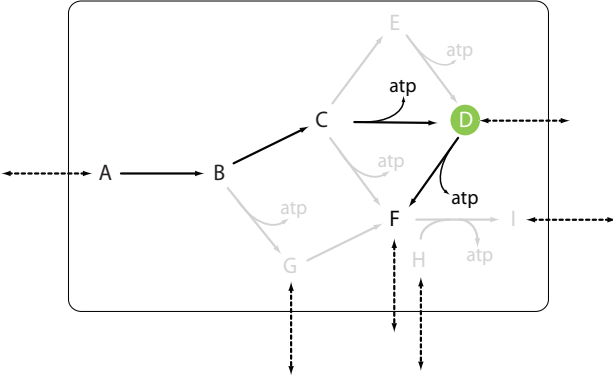

Random network 3

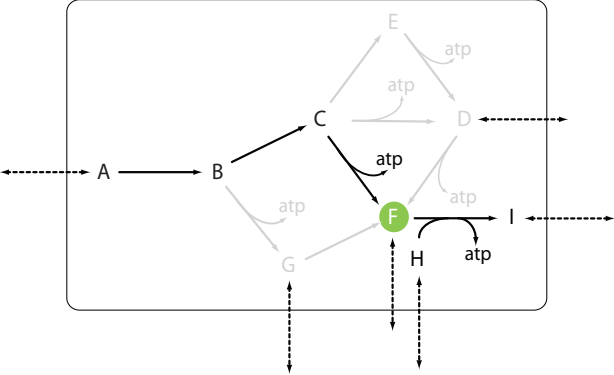

Random network 4

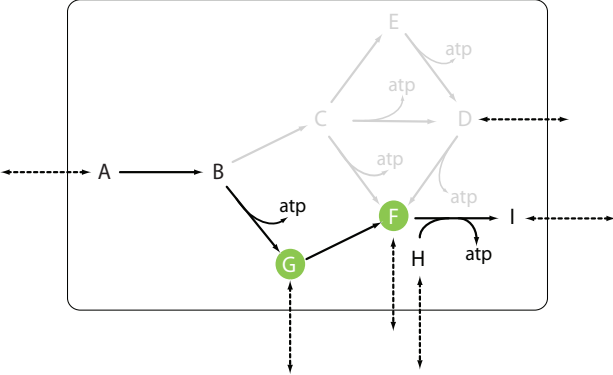

C

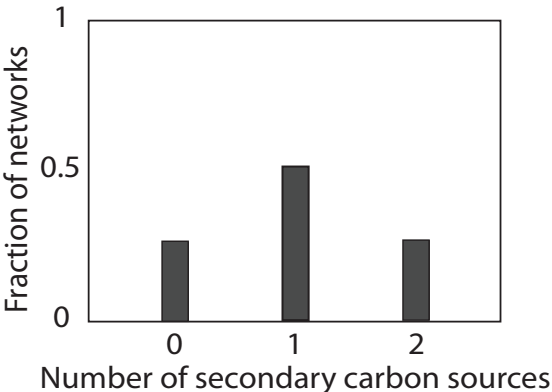

D

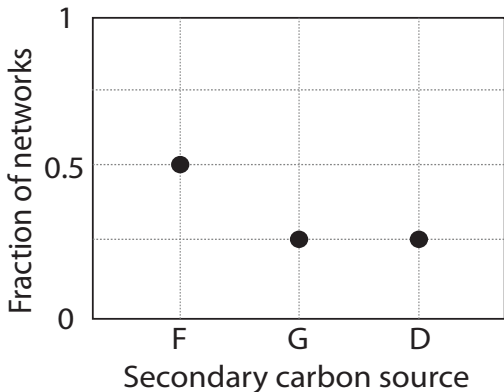

Supplement: S10 Fig — Panel (A) shows a hypothetical pan-metabolic network comprising 10 internal and 5 transport reactions. Panel (B) shows four networks created by randomly selecting 4 internal reactions (in black) from the pan-metabolic network. In this hypothetical example, ATP must be produced from at least one of the environmental nutrients (A, D, F and G) for a network to be viable. All four networks are viable on A if nutrient H is available in the environment. The random networks 2, 3 and 4 are also viable on metabolites D, F and F and G respectively. If a metabolite on which a network is viable can be produced when nutrient A is consumed, the metabolite is a secondary carbon source (green). (C) Histogram of the number of secondary carbon sources per random network, for the four random networks considered here. (D) Rank plot of secondary carbon sources (horizontal axis) that can be produced by at least one random network when A is used as a primary carbon source, ranked by the fraction of random viable networks (vertical axis) by which the secondary carbon source can be produced. (PDF) [file pcbi.1006340.s017.pdf]
