# Supplementary material for: An enormous potential for niche construction through bacterial cross-feeding in a homogeneous environment
Source: PLoS Comput Biol. 2018 Jul 24;14(7):e1006340. doi: 10.1371/journal.pcbi.1006340 (PMC6080805; doi:10.1371/journal.pcbi.1006340)
Supplement: S7 Fig — The outermost circle lists all of E. coli's carbon sources (as in Fig 2A), ordered clockwise according to biomass yield, starting from formate (for, 9’ o’clock). Green circles (solid green scale bar from center to top right) indicate the biomass yield of each carbon source. Black circles (solid black scale bar from center to top left) indicate the number of carbon atoms of the carbon source. Blue circles (solid blue scale bar from center to lower left) indicate the number of secondary carbon sources that can be produced when E. coli grows on a given primary carbon source. Red circles (solid red logarithmic scale bar from center to down right) indicate from how many primary carbon sources this carbon source can be produced as a secondary carbon source (if it can be produced at all). Most secondary carbon sources can be produced from all primary carbon sources (ln(179) = 5.2 on the red scale), but some can be produced only from few primary carbon sources. Among them is glucose (grey arrow), which can be produced only from four primary carbon sources. (PDF) [file pcbi.1006340.s014.pdf]

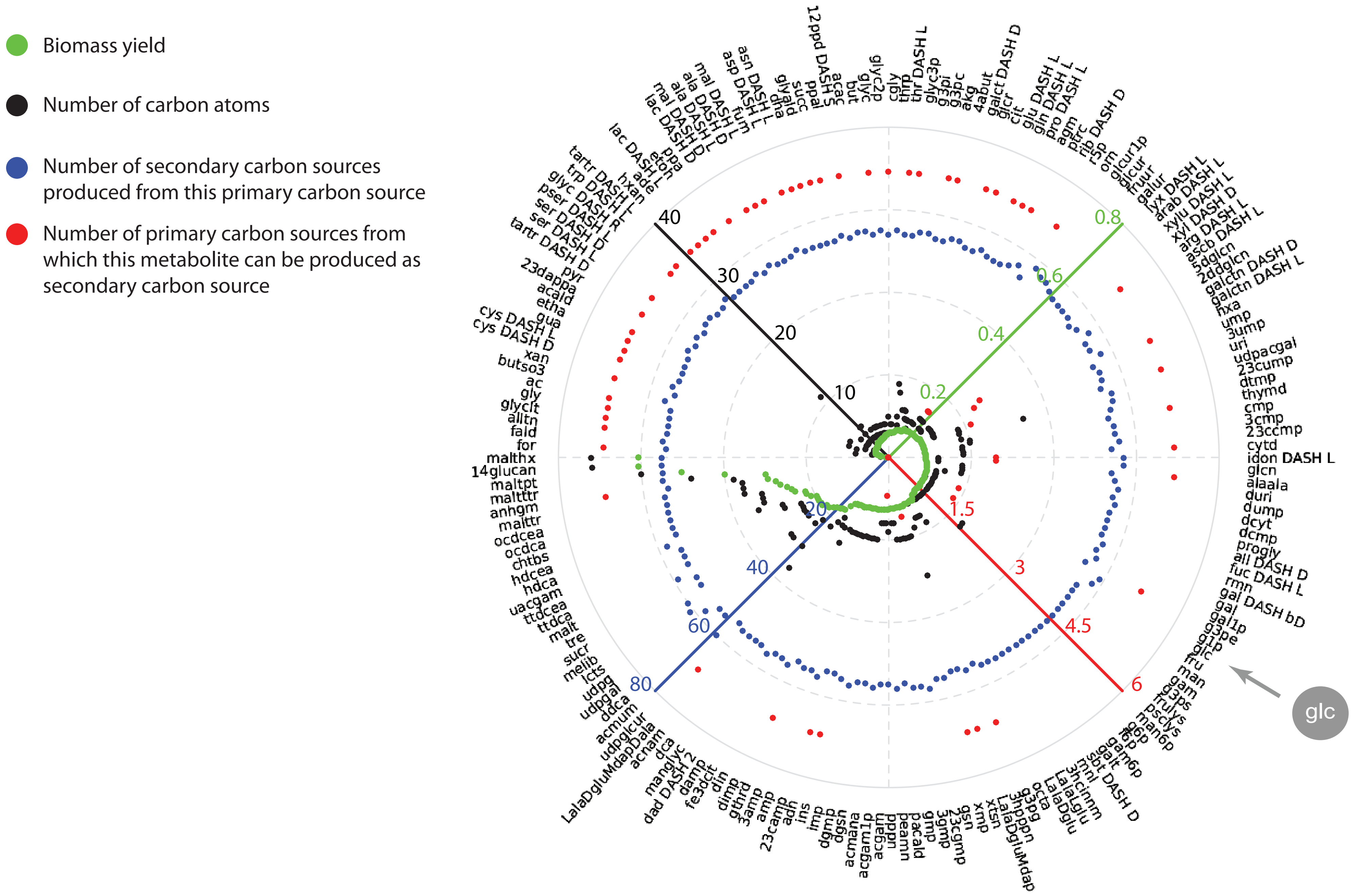

Biomass yield
Number of carbon atoms
Number of secondary carbon sources produced from this primary carbon source
Number of primary carbon sources from which this metabolite can be produced as secondary carbon source
10
20
30
40
0.2
0.4
0.6
0.8
20
40
60
80
1.5
3
4.5
6
glc
